# Long-Term Outcomes for Chinese COPD Patients After PCI: A Propensity Score Matched, Double-Cohort Study

**DOI:** 10.3389/fcvm.2022.827635

**Published:** 2022-06-09

**Authors:** Yitian Zheng, Yu Qi, Samuel Seery, Wenyao Wang, Wei Zhao, Tao Shen, Lequn Zhou, Jie Yang, Chen Li, Xuliang Wang, Jun Gao, Xiangbin Meng, Erdan Dong, Yi-Da Tang

**Affiliations:** ^1^Department of Cardiology, State Key Laboratory of Cardiovascular Disease, Fuwai Hospital, National Center for Cardiovascular Diseases, Chinese Academy of Medical Sciences and Peking Union Medical College, Beijing, China; ^2^Graduate School of Peking Union Medical College, Chinese Academy of Medical Sciences and Peking Union Medical College, Beijing, China; ^3^Department of Cardiology and Institute of Vascular Medicine, Peking University Third Hospital, NHC Key Laboratory of Cardiovascular Molecular Biology and Regulatory Peptides, Key Laboratory of Molecular Cardiovascular Science, Ministry of Education, Beijing Key Laboratory of Cardiovascular Receptors Research, Beijing, China; ^4^School of Humanities and Social Sciences, Chinese Academy of Medical Sciences and Peking Union Medical College, Beijing, China; ^5^Faculty of Health and Medicine, Division of Health Research, Lancaster University, Lancaster, United Kingdom; ^6^The Institute of Cardiovascular Sciences, Peking University, Beijing, China

**Keywords:** chronic obstructive pulmonary disease, coronary artery disease, percutaneous coronary intervention, outcomes, aging

## Abstract

**Objectives:**

The aim of this study was to analyze long-term outcomes of Chinese coronary artery disease (CAD) patients with (and without) chronic obstructive pulmonary disease (COPD) after percutaneous coronary intervention (PCI).

**Background:**

Chronic obstructive pulmonary disease is a chronic condition which often develops in conjunction with CAD. PCI is a core therapy for CAD, although we still need to understand CAD-COPD outcomes and to identify factors that influence prognoses, across ethnicities.

**Methods:**

This double-cohort study involved 12,343 Chinese CAD patients who received PCI. Baseline characteristics were collected in two independent, specialty centers. Propensity-score matching was performed to control confounding factors, using a nearest neighbor matching method within a 0.02 caliper and on a propensity score scale of 0.1 for each center. Comorbid CAD-COPD cases were compared to non-COPD patients in terms of major adverse cardiac events (MACEs).

**Results:**

Patients with COPD were generally older than those without COPD (65.4 ± 9.2 vs. 58.2 ± 10.3, *p* < 0.001). There were no significant differences in the end points between COPD and non-COPD groups after PCI (All *p* > 0.05); however, the incidence of MACEs increased after 450 days. Further subgroup analysis suggests that COPD is approximately four times more prevalent among those aged over 75 years (HR, 3.818; 95%CI, 1.10–13.29; *p* = 0.027) and those aged below 55 years (HR = 4.254; 95% CI, 1.55–11.72; *p* = 0.003).

**Conclusion:**

Having COPD does not appear to have a significant impact on CAD outcomes 2 years after PCI, and beyond. However, an increasing number of MACEs was observed after 450 days, which suggests that there may be a double-stage effect of COPD on PCI prognosis. There is a need for focused comorbidity management, specifically for those aged below 55 years and above 75 years.

## Introduction

Chronic obstructive pulmonary disease (COPD) is a chronic, progressive, and fatal condition, associated with substantial global mortality. COPD is related to various cardiovascular comorbidities (1–3) which has an impact on quality of life and longevity. The incidence of coronary artery disease (CAD) among COPD patients ranges from 2.4 to 23.3% (4–8). Aside from having these life-limiting conditions, CAD-COPD frequently encounters serious dyspnea, exercise intolerability, and edema (9) which complicate cardiac and pulmonary rehabilitation. Similarly, lifestyle factors including smoking status, diet, and aging are thought to play a casual role in the onset (and exacerbation) of symptoms (10, 11). Consequently, there are a number of questions about the effect of revascularization therapies on CAD-COPD patients.

Percutaneous coronary intervention (PCI) is a core therapy involved in coronary revascularization. Multiple studies have investigated COPD patient outcomes after receiving PCI therapies and have identified differences between ethnicities (12) and therefore lifestyles, but have also found that in-hospital prognosis and long-term outcomes are worse for those suffering from CAD-COPD (13–18). However, the average age of patients with COPD is generally higher than that of patients without COPD, which may be related to convenience sampling or factors involved in hospitalization and readmission. Likewise, different incidence rates are associated with distinct risk factors and, therefore, a number of issues remain around PCI prognosis and major adverse cardiac events (MACEs). Some studies do not appear to include cardiac death as a MACE and, indeed, despite spirometry being the diagnostic gold standard for COPD, many studies appear to utilize more subjective alternatives.

Given the aforementioned unknowns and such variability involved in diagnostics, there remains a need to conduct more rigorous research comparing spirometry or pulmonary function testing (PFT) for COPD diagnosis, despite seldom being used for CAD patients (19). It is possible to conduct retrospective research, although methods to control for confounding factors ought to be implemented. Propensity-score matching (PSM) is a statistical method of controlling the factors identified through previous research. The primary purpose of PSM was to estimate the treatment effect or interactions between two factors by intercalating covariates that may influence the outcomes (20). Understanding the relationship between CAD and COPD in patients who receive PCI may enable us to make more sophisticated comparisons and to further develop guidelines.

## Materials and Methods

### Study Design

**FIGURE 1:**
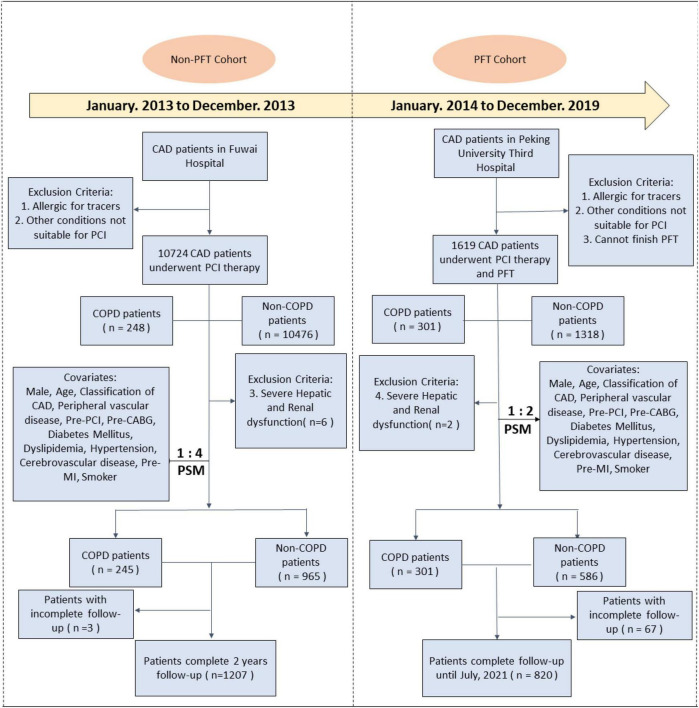
Study flowchart of the non-PFT and PFT cohorts in two centers. PFT, pulmonary function testing; CAD, coronary artery disease; PCI, percutaneous coronary intervention; COPD, chronic occlusion pulmonary disease; CABG, coronary artery bypass graft; MI, myocardial infarction.

As shown in Figure 1, consecutive CAD patients who received PCI in Fuwai Hospital, Beijing, were enrolled from 1 January to 31 December 2013. Fuwai Hospital did not (at that time) utilize PFT and was therefore referred to as the non-PFT cohort. Peking University Third Hospital adopted PFT and is hereafter referred to as the PFT cohort. Patients who received PCI in Peking University Third Hospital between 1 January 2014 to 31 December 2019 were enrolled.

Lab testing and data collection were conducted in the same manner for both cohorts. Controlling of confounders and statistical analysis of the two cohorts were performed independently, in accordance with predefined criteria and the methods described. Acute coronary syndrome (ACS) consists of three cardiac conditions, namely, ST elevation myocardial infarction (STEMI), non-ST elevation myocardial infarction (NSTEMI), and unstable angina (UA), according to guidelines (21). Chronic coronary syndrome (CCS) is defined by the manifesting phases of CAD, excluding situations in which an acute coronary artery thrombosis dominates the clinical presentation (22).

Ethical approval was obtained through Fuwai Hospital Research Ethics Committee and Peking University Third Hospital Research Ethics Committee. The respective Institutional Review Boards approved the protocol and all patients were provided with information regarding the study and our objectives, before providing informed consent.

### Participants

All participants received PCI, although the procedural method was dependent on specific operators. Participants who have allergies to tracers and other conditions were excluded, given that they could not receive PCI. Those with severe hepatic and renal dysfunction (severe hepatic dysfunction: serum bilirubin ≥ 5 mg/dl (85 mmol/L) and coagulopathy (INR ≥ 1.5 or prothrombin activity < 40%); severe renal dysfunction: GFR less than 30 ml/min/1.73 m^2^) were considered at an extremely higher risk of MACEs, and were therefore also excluded in this study.

### Non-pulmonary Function Testing Cohort

A total of 10,724 patients with CAD who had received PCI in Fuwai Hospital were enrolled in the non-PFT cohort. Patient characteristics, information around co-existing conditions, and procedural details were collected independently by at least two physicians. Participant data were then assigned to either the COPD or non-COPD subgroup. COPD diagnosis was based on patients’ clinical histories checked by International Classification of Diseases, 9th edition (ICD-9) coding 496 (COPD) on admission, combined with the presentation of symptoms, treatment specific for COPD, and imaging. CAD diagnosis was also standardized based on coronary angiography.

### Pulmonary Function Testing Cohort

A total of 1,619 CAD patients who had received PCI and PFT in Peking University Third Hospital between 1 January 2014 and 31 December 2019 were assigned to the PFT cohort. Again, patients’ baseline characteristics, co-existing conditions, and procedural details were collected independently by at least two physicians. In the PFT cohort, COPD was defined as a post-bronchodilator forced expiratory volume in the 1st second (FEV1)/forced vital capacity (FVC) ratio of 0.70.

### Treatment

Before receiving selective PCI, patients who had not taken long-term aspirin and a P2Y12 inhibitor, received 300 mg aspirin and a P2Y12 inhibitor with a loading dose. Patients, who were scheduled for primary PCI, received the same dose of aspirin and clopidogrel with a loading dose of 300 mg or 600 mg, according to bleeding risk.

During PCI, 50–100 U/kg of heparin sodium was administered according to the bleeding risk. From the vision of CAG (coronary angiography), more than 50% stenosis of the left main artery (LM), left anterior descending artery (LAD), left circumflex artery (LCX), right coronary artery (RCA), and the main branch of these vessels were defined as coronary artery stenosis. Patients with greater than 70% stenosis in vessels and ischemic symptoms were recommended for coronary stent implantation.

### Follow-Up

Participants were visited after 30 days, 6 months, and each year after PCI. Follow-up in the non-PFT cohort was completed in 2 years. Follow-up in the PFT cohort was completed in July 2021. Primary end points were MACEs, consisting of cardiac death, unplanned revascularization (further referred to as simply revascularization), and non-fatal myocardial infarction (MI). Secondary end points included components of MACEs, all-cause death, stroke, and device-oriented composite end point (DOCE). Outpatient follow-up was completed by cardiologists in both centers. For those unable to return to the hospital, follow-up was completed over the telephone by clinical workers and clinical research coordinators.

### Propensity Score Matching

Propensity score matching (PSM) was implemented before statistical analysis to achieve balanced exposure at the baseline for specific covariates that may affect outcomes. These covariates included age, gender, type of CAD, and other coexisting conditions described in Figure 1. Participants’ prognosis and other aggregated data are provided in the Supplementary Tables 1, 2.

Given the comparatively large number of participants in the non-PFT cohort, a nearest-neighbor matching procedure was adopted within a caliper of 0.01 on the propensity score scale. COPD patients were matched to non-COPD patients at a ratio of 1:4 in the non-PFT cohort. Nearest-neighbor matching was also adopted within a caliper of 0.1 and at a ratio of 1:2 in the PFT cohort. Baseline characteristics of COPD and non-COPD subgroups were compared before and after PSM for scientific rigor. Variables with standardized differences < 10% between the two groups were considered well-balanced.

### Statistical Analysis

This double-cohort study was designed to determine whether COPD is detrimental for CAD patients who received PCI at 2 years and beyond. Continuous variables are presented as means with corresponding standard deviations (SD), while categorical data are presented as simple numbers with percentages.

Mann–Whitney’s U-test and Student’s *t*-test were implemented to compare continuous variables. Standard chi-square tests were used to compare categorical variables. Cumulative event rates for each cohort were measured using Kaplan–Meier curves and two-sided Log-rank tests. In instances where the requirements of proportional hazard for survival analysis were not met, Cox’s survival analysis was not considered. Hazard ratios (HRs) with corresponding 95% confidence interval (CI) were calculated using the Mantel-Cox method. Possible confounding factors in our study include age, male, CAD classification, hypertension, dyslipidemia, diabetes mellitus, renal dysfunction, smoking, cerebrovascular diseases, previous myocardial infarction, previous CABG, previous PCI, and peripheral vascular disease.

Landmark analysis was conducted according to the interactions between Kaplan–Meier curves when necessary. In this study, the landmark point was set at 450 days, for both non-PFT and PFT cohorts, with HRs being calculated separately for events that occurred up to 450 days, after PSM, and events that occurred between 450 days and the end of the follow-up period.

For further exploratory subgroup analysis of the PFT cohort, participants were further separated according to predefined covariates mentioned previously, of which some covariates were excluded due to the limited number of participants. A subgroup with HRs and *p*-values and *p*-values for interactions less than 0.1 were considered in the constructed effect modification model. Possible risk factors were intercalated to determine the relationship between different age stratifications and COPD in the long-term outcome of CAD patients who underwent PCI. *P*-values less than 0.05 were considered statistically significant.

Considering that the total number of participants in the PFT cohort was less than 1,000, with 301 participants in the COPD group, the marginal significance was set at *p* ≤ 0.1 for the end point incidence rate and related HRs (23).

All statistical analyses were performed by two investigators (YZ at Fuwai, Hospital and YQ at Peking Third Hospital) with guidance from a clinical epidemiologist (SS). SPSS (version 26.0), Graphpad Prism (version 8.0), and Rstudio (Rversion 4.0) were used to perform all statistical analyses.

## Results

### Patient Follow-Up

Between January and December 2013, 10,724 consecutive CAD participants who underwent PCI were enrolled in the non-PFT cohort. After PSM, 1,207 (99.8%) participants completed the 2-year follow-up in the non-PFT cohort. The mean for the duration of follow-up was 794 (SD = 243) days for patients after the first-time of PCI in the non-PFT cohort.

Between January 2014 and December 2019, 1,619 consecutive CAD participants underwent PCI and PFT were enrolled in the PFT cohort. After PSM, 820 (92.4%) participants completed the follow-up until July 2021. The mean for the duration of follow-up was 1,300 (SD = 609) days for patients after the first-time PCI in the PFT cohort, with the longest follow-up of 2,774 days.

### Baseline Characteristics

As shown in the Supplementary Table 1, there were 248 patients in the COPD group and 10,476 patients in the non-COPD group before PSM in the non-PFT cohort. After PSM, the number of patients in the COPD and the non-COPD groups were more comparable, i.e., 245 vs. 965. Before PSM, the patients in the COPD group were older than those in the non-COPD group, 65.4 (SD = 9.2) years vs. 58.2 (SD = 10.3) years, standardized difference = 78.4%; *p* < 0.001, with increased prevalence of renal dysfunction (6.0% vs. 3.4%, standardized difference = 11.2%; *p* = 0.023), cerebrovascular diseases (16.5% vs. 10.6%, standardized difference = 16%; *p* = 0.003), and more peripheral vascular diseases (12.5% vs. 7.5%, standardized difference = 19%; *p* = 0.003). After PSM, age in the COPD group and non-COPD group was nearly equal with a similar distribution in different stages, 65.3 (SD = 9.1) years as opposed to 64.6 (SD = 9.9) years; standardized difference = 7.2%; *p* = 0.257.

Classification of CAD is nearly the same in the COPD group and non-COPD group (CCS: 9.8% vs. 9.9, ACS: 90.2% vs. 90.1%, standardized difference = 0.6%; *p* = 0.943) after PSM. Overall, standardized differences under all coexisting conditions are less than 10% after PSM. For lab tests and procedure details, the albumin level is slightly decreased in the COPD group before and after PSM (41.4 ± 4.2 vs. 42.3 ± 4.2, *p* = 0.002).

Lab tests, including urine acid, LDL-C (low density lipoprotein-cholesterol), LVEF (left ventricular eject fraction), and creatine, were similar in the COPD and non-COPD groups with no statistical significance (all *p*-values > 0.05). After PSM, procedure details in the COPD and non-COPD groups were almost equivalent (*p* > 0.05).

As shown in the Supplementary Table 2, there were 301 patients in the COPD group and 1,318 patients in the non-COPD group before PSM in the PFT cohort. After PSM, the number of patients in the COPD and non-COPD groups is almost equal (301 vs. 586). Before PSM, patients in the COPD group are older than the non-COPD group (62.8 ± 9.3 vs. 59.1 ± 10.5, standardized difference = 36.6%; *p* < 0.001), with more smokers (66.1% vs. 57.1%, standardized difference = 18.9%; *p* = 0.004). After PSM, age in the COPD and non-COPD group is comparative, with a similar mean age and distribution at different stages (62.8 ± 9.3 vs. 61.9 ± 10.1, standardized difference = 2.3%). Classification of CAD is nearly the same in the COPD and non-COPD groups (CCS: 32.9% vs. 32.6%, ACS: 67.1% vs. 67.4%, standardized difference = 1.1%) after PSM. Overall, standardized differences under all coexisting conditions are less than 10% after PSM.

### Primary End Points

**FIGURE 2:**
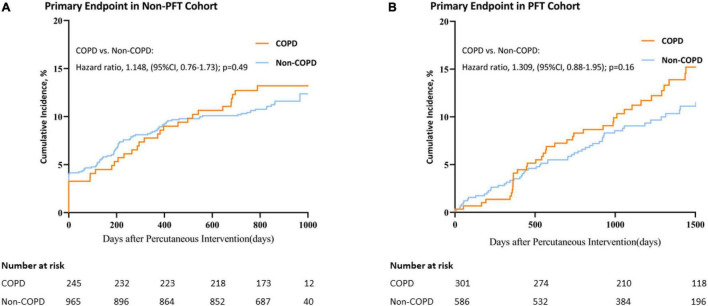
Kaplan–Meier curves for primary end point in the non-PFT and PFT cohorts. Plot of Primary end point Incidence for COPD vs. non-COPD. **(A)** Primary end point in the non-PFT Cohort; **(B)** Primary end point in the PFT cohort. CI, confidence interval.

In the non-PFT cohort, although the percentage of patients who had a primary end point event is higher in the COPD group than in the non-COPD group at 2 years, it cannot be considered significant (13.1% vs.11.3%; non-COPD HR, 1.148; 95% CI, 0.76 to 1.73; *p* = 0.49) (see Figure 2 and Table 1 for further details).

**TABLE 1:** Long-term outcomes after PCI in the non-PFT and PFT Cohorts after PSM.

	Non-PFT cohort			PFT cohort		
Endpoints	No. of Events,%	Harzard Ratio (95% confidence interval)	*P*-value	No. of Events,%	Harzard Ratio (95% confidence interval)	*P*-value
***Primary Endpoint***	141			107		
COPD	32 (13.1)	1.148 (0.76–1.73)	0.49	44 (14.6)	1.309 (0.88–1.95)	0.16
Non-COPD	109 (11.3)	0.871 (0.58–1.31)		63 (10.8)	0.764 (0.51–1.14)	
***Components of primary endpoint***						
**Cardiac death**	11			5		
COPD	3 (1.2)	1.481 (0.34–6.46)	0.56	1 (0.3)	0.481 (0.08–3.05)	0.5
Non-COPD	8 (0.8)	0.675 (0.15–2.94)		4 (0.6)	2.081 (0.33–13.20)	
**Myocardial infarction**	55			7		
COPD	9 (3.7)	0.77 (0.39–1.48)	0.54	3 (0.9)	1.417 (0.30–6.72)	0.65
Non-COPD	46 (4.8)	1.302 (0.67–2.51)		4 (0.7)	0.706 (0.15–3.35)	
**Revascularization**	84			100		
COPD	20 (8.2)	1.216 (0.72–2.07)	0.45	42 (14.0)	1.355 (0.89–2.04)	0.13
Non-COPD	64 (6.6)	0.823 (0.48–1.40)		58 (9.9)	0.738 (0.49–1.11)	
***Device-oriented composite endpoint***	50			57		
COPD	11 (4.5)	1.103 (0.55–2.20)	0.78	25 (8.3)	1.468 (0.85–2.53)	0.14
Non-COPD	39 (4.0)	0.906 (0.46–1.80)		32 (5.6)	0.681 (0.40–1.18)	
***All-cause death***	31			24		
COPD	8 (3.3)	1.365 (0.57–3.28)	0.45	12 (4.0)	1.876 (0.81–4.34)	0.11
Non-COPD	23 (2.4)	0.732 (0.31–1.76)		12 (2.0)	0.533 (0.23–1.24)	
***Stroke***	27			11		
COPD	6 (2.4)	1.111 (0.44–2.83)	0.82	3 (0.9)	0.69 (0.20–2.38)	0.58
Non-COPD	21 (2.2)	0.900 (0.35–2.29)		8 (1.4)	1.447 (0.42–4.99)	

*MACE, major adverse cardiovascular events; COPD, chronic occlusion pulmonary disease; PFT, pulmonary function testing; PCI, percutaneous coronary intervention; PSM, propensity score matching; Device-oriented composite end point contains Cardiac death, target vessel myocardial infarction, and target lesion revascularization.*

In the PFT cohort, the percentage of patients who had a primary end-point event is not seen to be significantly higher in the COPD group than in the non-COPD group with a longer follow-up than the non-PFT cohort (14.6% vs.10.8%; non-COPD HR, 1.309; 95% CI, 0.88 to 1.95; *p* = 0.16) (see Figure 2 and Table 1 for further details).

No statistically significant difference was observed in both non-PFT and PFT cohorts. However, interactions between discovery and validation curves could be observed when follow-up time approached 450 days (Figure 2). This suggests that the proportional hazard assumption was not suitable in these two cohorts or for long-term outcomes after PCI.

**FIGURE 3:**
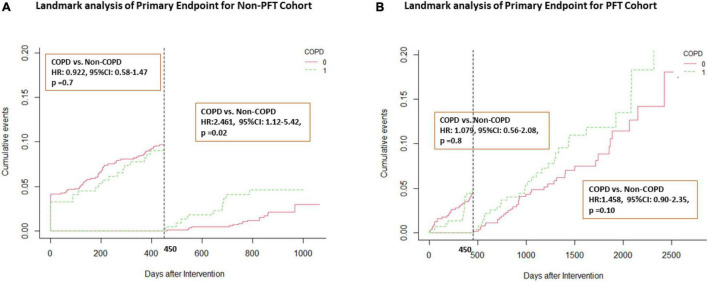
Landmark analysis of primary end point in both cohorts. Landmark curve according to a time point of 450 days. **(A)** Landmark analysis in the non-PFT cohort; **(B)** Landmark analysis in the PFT cohort. HR, hazard ratio; CI, confidence interval.

Figure 3 shows the results from the landmark analyses of the primary end point in both the discovery and PFT cohorts. Before 450 days, the COPD group has a similar HR to the non-COPD group, all with *p*-values > 0.05 in both the non-PFT and PFT cohorts. After 450 days, the COPD group HR increased with the non-COPD group in the non-PFT cohort (non-COPD HR, 2.461; 95% CI, 1.12 to 5.42; *p* = 0.02) (see Figure 3A for further details). In the PFT cohort, the COPD group also exhibits a higher incidence rate of MACE after 450 days (non-COPD HR, 1.458; 95% CI, 0.90 to 2.35; *p* = 0.10) (see Figure 3B).

### Secondary End Points

In the Supplementary Figures 1, 2 show the secondary end points of the non-PFT cohort and PFT cohort as Kaplan–Meier curves. No end points differed significantly between the COPD and non-COPD groups, regardless of the follow-up period. In Supplementary Figures 3–8 present comparative landmark analyses (non-PFT and PFT) for secondary end points through KM curves with a landmark point of 450 days.

The number of deaths increased in both non-PFT cohort and PFT cohort (non-PFT cohort HR, 2.461; 95% CI, 1.12–5.42; *p* = 0.02; PFT cohort HR, 2.386; 95%CI, 0.89–6.41; *p* = 0.08) (see Supplementary Figure 3). As a component of the primary end point, unplanned revascularization was higher in the COPD group after 450 days, with a marginal significance (hazard ratio in the non-PFT cohort, 2.489; 95% CI, 0.96–6.42; p = 0.05; hazard ratio in the PFT cohort, 1.473; 95%CI, 0.90–2.42; *p* = 0.09) (see Supplementary Figure 6 for further details). Before 450 days, no significant difference in the secondary end points was observed which differed significantly in the COPD group and non-COPD group among both the non-PFT cohort and PFT cohort. The DOCE was also increased in both the non-PFT cohort and PFT cohort after 450 days, with a marginal significance (hazard ratio in the non-PFT cohort, 2.35; 95% CI, 0.85–6.47; p = 0.09; hazard ratio in the PFT cohort, 1.58; 95%CI, 0.93–2.85; p = 0.07).

### Exploratory Subgroup Analysis

**FIGURE 4:**
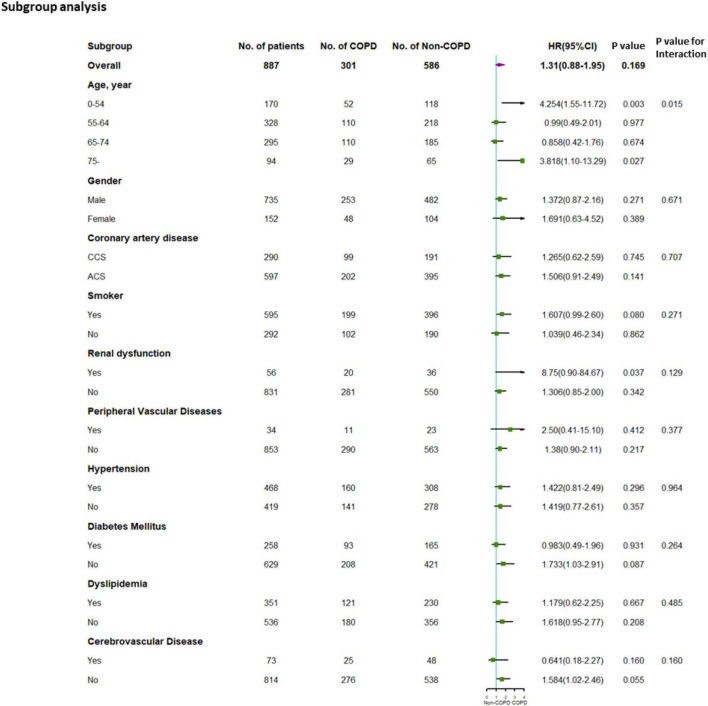
Subgroup analysis for the PFT cohort. Subgroup analysis performed according to possible covariates. CCS, chronic coronary syndrome; ACS, acute coronary syndrome. HR, hazard ratio.

In the PFT cohort, exploratory subgroup analyses were performed according to the covariates described above. As shown in Figure 4, age exerts a detrimental impact on the prognosis of COPD vs. non-COPD (*p*-value for interaction = 0.015). Among age less than 55 years of the subgroup and age over 75 years of the old subgroup, COPD exerts an obviously worse outcome after PCI (age less than 55 years: HR, 4.254; 95%CI, 1.55–11.72; *p* = 0.003; age over 75 years: HR, 3.818; 95%CI, 1.10–13.29; *p* = 0.027).

To avoid false-positive errors in the subgroup analysis, effect modification of age on long-term outcomes of patients after PCI with or without COPD was then carried out combining with possible confounding factors during subgroup analysis according to different end points (Supplementary Table 3).

For MACEs, the adjusted HR was significantly different in the COPD group vs. the non-COPD group in patients aged over 75 years (adjusted HR: 3.772, adjusted 95% CI 1.07–13.30, and adjusted *p* = 0.039). The adjusted *p*-value for interaction indicated marginal statistical significance in the interaction of age over 75 years to COPD (MACE: adjusted *p* for interaction = 0.071) (see Supplementary Table 3 for further details).

For patients aged less than 55 years, the adjusted HR of the MACE significantly differed in the COPD group and non-COPD group, which was mainly driven by unplanned revascularization (adjusted HR: 4.295, adjusted 95% CI: 1.56–11.86, adjusted *p* = 0.005). The adjusted *p*-value for interaction indicated statistical significance in the interaction of age less than 55 years in COPD (MACE: adjusted *p* for interaction = 0.018) (see Supplementary Table 3 for further details).

Further end point analysis was conducted according to various GOLD levels. It was performed to explore the impact of COPD severities on prognosis after PCI. MACEs and DOCEs were observed and increased when comparing GOLD III/IV to GOLD I/II, although no statistically significant differences were found (see Supplementary Table 4 for details).

## Discussion

The aim of this study was to analyze CAD patients with and without COPD, in order to identify factors that may influence prognoses after PCI. Understanding the relationship between CAD and COPD in those who received PCI may help us gain insight into differences within and between populations and also help develop clinical guidelines. A total of 12,343 participants were enrolled from two specialty units in Beijing and were followed-up for a period of 4 years. Those with COPD appeared to be significantly older, with a greater chance of renal dysfunction, and cerebro- and peripheral vascular diseases. After propensity-score matching, the differences across all coexisting conditions were less than 10%.

There were procedural differences between the non-PFT cohort and PFT cohort. In the non-PFT cohort, patients were diagnosed as having COPD through clinical history, presentation of symptoms, combined imaging, and COPD management techniques. These techniques are obviously more subjective, which means that there would be diagnostic differences, given such variability in clinical practice (24). Spirometry is not necessarily expensive although physicians may think of it as rather time consuming, generally taking 30–60 min. Some physicians may also encounter difficulties interpreting spirograms, which may relate to confidence in both using and interpreting spirograms (25). In the United Kingdom, there are some institutes which have PFT technologies, yet only 85.6% of all practices use spirometry (25). Therefore, there may be a need for training to ensure that physicians are more confident using spirograms correctly because PFT can provide information to differentially diagnose acute bronchitis and interstitial lung disease.

Inclusion criteria for the PFT cohort were more precise than those set in place for the non-PFT cohort. Diagnosing COPD in CAD patients without spirometry can lead to misdiagnosis (18, 26, 27). In the PFT cohort, patients were diagnosed through spirograms in combination with patient histories. Therefore, the PFT cohort provides more reliable evidence, which better reflects the impact of COPD on CAD patients who received PCI. The PFT cohort consisted of only 1,619 patients which may be the result of more accurate diagnostic methods but some patients may have been excluded unnecessarily. Like pulmonary diseases, CADs are not a homogeneous set of diseases, meaning that there may have been patients who suffered a form of CAD but were unable to complete PFT due to suspected heart failure or another related cardiopulmonary condition.

These issues all are related to our ability to differentially diagnose both CADs and COPD. Presently, CAD diagnosis is complicated by the availability of testing, technologies, and increasingly, costs (28). We know the risk factors and the causes, but there are also a number of complications such as heart failure, arrhythmias, and diabetes. Each of these related factors influences not only diagnosis but also treatments and therefore prognosis. Fortunately, there are a number of novel diagnostic methods coming into clinical practice. For example, through bioinformatics analysis, Zhang et al. (29) have managed to identify several biomarkers that could potentially be used to differentially diagnose CADs in terms of genetic signatures (29). Likewise, Jing et al. have found that the levels of expression of Homer 1, IL-1β, and TNF-α may be used to differentially diagnose CADs (30), meaning that there are opportunities on the horizon which will improve diagnosis beyond spirometry. Although, comparative retrospective studies and indeed clinical trials are needed.

The primary end points in this study were MACEs which included cardiac death, revascularization, and non-fatal myocardial infarction. There was no significant difference in the incidence of MACEs in patients with COPD compared to those who did not have COPD, after PSM. This finding is not consistent with previously published studies. For example, according to the BASKET-PROVE I and II trials, COPD is associated with increased 2-year rates of MACEs (8). Researchers also found that cardiac death was most common, whereas target vessel revascularization and non-fatal myocardial infarction did not appear to increase significantly (8). However, subgroup analyses in the BASKET-PROVE trials were based on the effects of different types of stents, i.e., bare metal vs. drug-eluting stents, which were not adjusted for when calculating HRs for cardiac death. Sung et al. also reported that having COPD is not an independent predictor of short-term or medium-term MACOs, i.e., major adverse clinical outcomes (death, recurrent myocardial infarction, or re-admission for congestive heart failure) in patients with STEMI (ST-segment elevation myocardial infarction) undergoing primary PCI (5). Januszek et al. reported increasing periprocedural complications, such as restenosis and in-stent thrombosis, in patients with COPD and PCI (31, 32). However, end points among these different studies may partly explain why the conclusions differ. In this study, we considered MACEs that are commonly used in cardiovascular studies, as a fixed end point.

In this study, data revealed a two-stage survival curve in both the non-PFT and PFT cohorts. Even though COPD was not eventually seen to worsen the long-term outcomes for CAD patients after PCI, a bidirectional effect was considered before and after 450 days. An increasing number of MACEs were observed after 450 days in both non-PFT and PFT cohorts. Possible explanations may be related to the characteristics of COPD itself, such as onset age. There is, of course, an overlap between COPD and CAD in terms of symptoms, such as inflammation, airflow limitations, and increased platelet activation (33–35). Also, PCI is an invasive therapy and patients always receive dual anti-platelet therapy after PCI in accordance with guidelines (36). Anti-platelet levels downregulate after 1 year due to bleeding concerns (37–39), leaving patients in a potentially hypercoagulable state. Likewise, platelet activation has been implicated in COPD (40). Therefore, we suspect insufficient anti-platelet therapy after 1 year for patients with concomitant CAD-COPD which may cause an increased likelihood of MACEs. Another explanation may be that restricted airflow and inflammation are both caused by and have a catalytic effect on the course of CAD-COPD progression. This certainly requires further physiological research and longer-term clinical trials to explore these interactions.

In this study, we did not observe a significant difference in end points between the COPD and non-COPD subgroup, regardless of the follow-up period. However, multiple studies have reported an increased mortality in COPD patients after PCI (13, 27, 41). Our findings may not be consistent with previous findings, because we balanced ages after PSM. This approach is thought to influence COPD mortality readings (42, 43); however, an increasing number of deaths and unplanned revascularizations were observed after 450 days in both cohorts. This may have occurred as a result of COPD progression in aging CAD patients, although we also found that an increasing number of unplanned revascularizations positively correlates with an increase in MACEs after 450 days in both cohorts. However, this finding can only be considered of borderline significance (*p* = 0.05 in the non-PFT cohort; *p* = 0.09 in the PFT cohort). This may due to relatively limited numbers of participants in the COPD group. We also conducted a further comparison of GOLD levels according to prognosis after PCI. We observed an increased number of MACEs and DOCEs in GOLD III/IV groups, although this was not statistically significant. This may be due to small samples across each of the GOLD groups. Therefore, the impact of pulmonary function on CAD prognosis requires further research.

As previously mentioned, one factor that might worsen COPD and CAD patient outcomes is increased platelet activation (35, 40, 44). The increased number of revascularizations in the COPD group obviously requires appropriate management with anti-platelet therapies, which may lower the need for revascularizations but further research is required. One can assume that aging plays an important role in the progression and outcome of patients with COPD and CAD. In an attempt to understand this further, we conducted exploratory subgroup analysis. After conducting this analysis, we found that age and renal dysfunction appear to exacerbate symptoms and worsen outcomes for COPD patients who received PCI. Considering the imbalance in baseline characteristics after subgroup analysis, we decided to further explore interactions. Further *p*-values for interaction with renal dysfunction indicated no statistical differences, which means that these limited samples may have led to false-positive results. Age remained important for all patients after PCI. For COPD patients, aging stifles pulmonary functions, encourages inflammation, and increases vascular endothelium damage (9, 45). This may also explain why those aged above 75 years with COPD have quadrupled the risk compared to those without COPD. In this study, aging seems to interact with COPD, which worsens prognosis for patients after PCI. We supposed that the possible enlarged inflammation in elder COPD patients may play a pivotal role. Another possible reason may be the increased vascular endothelium damage, which may lead to a higher risk of platelet over-activation. Exacerbated pulmonary function may finally cause pulmonary heart disease, which in turn causes an imbalance of oxygen (supply and demand), and lead to an increased number of MACEs.

We also found that for patients aged below 55 years, COPD dramatically affects prognosis after PCI. Recently, Chayakrit et al. and Zuhdi et al. reported possible risk factors for young CAD patients using different databases; although their findings varied, smoking was a consistent risk factor (46, 47). Smoking is also known to be casual in the onset of COPD and correlates with a younger onset age. However, according to recent basic research, there is a genetic contribution at the early stage of COPD (48). Through our study, young CAD-COPD patients were at substantially higher risk compared to CAD patients. There are a number of different reasons for this finding. However, it is beyond the remit of this report to discuss this in detail. Hence, this finding supports the need for increased public health campaigns focusing on smoking prevention and cessation. There is also a need for more strict national policies to control air pollution, which is increasingly shown to be involved in respiratory and cardiovascular diseases. The approaches implemented should (according to these findings) focus on younger or elder adults. Again, habitual behaviors and lifestyles are issues that are more commonly discussed although China is a vast country with a complex, multilayered, and disparate set of cultures. Therefore, further studies ought to implement mixed methods, from interdisciplinary perspectives. Other studies (beyond lifestyle and behaviors) could focus on monitoring cardiac rhythms, preventing pulmonary cardiac dysfunction, and the standard use of β-blockers, especially for elderly patients. However, related research is lacking and requires our attention.

Before making any recommendations, we should consider the limitations of this study. In this instance, it was only possible to analyze and report findings based on a 2-year follow-up for the non-PFT cohort and 4 years for the PFT cohort. Following-up 10,000 plus patients over a period of 5 years is incredibly labor-intensive. However, research is ongoing and we shall report findings to share knowledge and encourage learning. There is also a need to further analyze those in the PFT cohort in terms of false positives and false negatives for both CADs and COPD. This will ensure that appropriate comparisons are made to help develop guidelines for the global population. Furthermore, more information about COPD and CAD patients, including CAT scores, MRC dyspnea scores, and the therapeutic methods implemented for COPD, will be recorded in the future. This is due to our assessment of COPD severities through these models, which may also help us to gain knowledge about interactions between COPD and CAD. Further research into therapies for patients with different COPD severities is necessary.

## Conclusion

Exacerbations are associated with these comorbid systemic disorders; however, having COPD does not appear to have a statistically significant impact on Chinese CAD patients 2 years after PCI, and beyond. There was also no significant difference in MACE incidence across the COPD group compared to the non-COPD group. However, an increase was observed after 450 days which highlights the need for focused comorbidity management methods. Patients aged above 75 years and below 55 years, who received PCI, appear to be at an increased risk of encountering MACEs which may be related to normal physiological deterioration or lifestyles although further research is required.

## Data Availability Statement

The raw data supporting the conclusions of this article will be made available by the authors, without undue reservation.

## Ethics Statement

The studies involving human participants were reviewed and approved by the Fuwai Hospital Research Ethics Committee and Peking University Third Hospital Research Ethics Committee. The patients/participants provided their written informed consent to participate in this study.

## Author Contributions

YZ, YQ, and Y-DT designed the study. WZ, TS, WW, LZ, JY, CL, XW, JG, and XM performed the clinical investigations and collected the data. YZ, YQ, and SS performed the data analysis and wrote the manuscript. YZ, YQ, SS, Y-DT, and ED revised the manuscript. All authors contributed to the article and approved the submitted version.

## Conflict of Interest

The authors declare that the research was conducted in the absence of any commercial or financial relationships that could be construed as a potential conflict of interest. The reviewer ZT declared a shared affiliation with the authors YZ, JY, CL, and XW to the handling editor at the time of review.

## Publisher’s Note

All claims expressed in this article are solely those of the authors and do not necessarily represent those of their affiliated organizations, or those of the publisher, the editors and the reviewers. Any product that may be evaluated in this article, or claim that may be made by its manufacturer, is not guaranteed or endorsed by the publisher.

## Abbreviations

COPD: chronic obstructive pulmonary disease
CAD: coronary artery disease
PCI: percutaneous coronary intervention
MACE: major adverse cardiac events
PSM: propensity-score matching
PFT: pulmonary function testing
FEV1: forced expiratory volume in the 1st second
FVC: forced vital capacity.
