# Supplementary material for: Long-Term Outcomes for Chinese COPD Patients After PCI: A Propensity Score Matched, Double-Cohort Study
Source: Front Cardiovasc Med. 2022 Jun 9;9:827635. doi: 10.3389/fcvm.2022.827635 (PMC9218100; doi:10.3389/fcvm.2022.827635)
Supplement: Supplementary file 1 [file Data_Sheet_1.docx]

**Supplementary materials**

**Supplementary Figure S1.** Kaplan-Meier curves of secondary endpoint in non-PFT cohorts

**Legend:** A. Death; B. Cardiac Death; C. Non-fatal Myocardial Infarction; D. Unplanned Revascularization; E. Stroke; F. DOCE. All p > 0.05

**Supplementary Figure S2.** Kaplan-Meier curves of secondary endpoint in PFT cohort

**Legend:** A. Death; B. Cardiac Death; C. Non-fatal Myocardial Infarction; D. Unplanned Revascularization; E. Stroke; F. DOCE. All p > 0.05

**Supplementary Figure S3.** Landmark analysis of deaths in double cohorts

**Legend: A.** Landmark analysis of deaths in Non-PFT cohort; **B.** Landmark analysis of deaths in PFT cohort. HR = Hazard Ratio; CI = Confidence Interval

**Supplementary Figure S4.** Landmark analysis of cardiac death in double cohorts **Legend: A.** Landmark analysis of cardiac death in non-PFT cohort; **B.** Landmark analysis of cardiac death in PFT cohort. HR = Hazard Ratio; CI = Confidence Interval

**Supplementary Figure S5.** Landmark analysis of non-fatal myocardial infarction in double cohorts

**Legend: A.** Landmark analysis of non-fatal myocardial infarction in non-PFT cohort; **B.** Landmark analysis of non-fatal myocardial infarction in PFT cohort. HR = Hazard Ratio; CI = Confidence Interval

**Supplementary Figure S6.** Landmark analysis of unplanned revascularization in double cohorts

**Legend: A.** Landmark analysis of unplanned revascularization in non-PFT cohort; **B.** Landmark analysis of unplanned revascularization in PFT cohort. HR = Hazard Ratio; CI = Confidence Interval

**Supplementary Figure S7.** Landmark analysis of stroke in double cohorts

**Legend: A.** Landmark analysis of stroke in non-PFT cohort; **B.** Landmark analysis of Stroke in PFT cohort. HR = Hazard Ratio; CI = Confidence Interval

**Supplementary Figure S8.** Landmark analysis of DOCE in double cohorts

**Legend: A.** Landmark analysis of stroke in non-PFT cohort; **B.** Landmark analysis of stroke in PFT cohort. HR = Hazard Ratio; CI = Confidence Interval

| \| **Supplemental Table S1. Baseline characteristics before and after Propensity-Score Matching in Non-PFT Cohort** \| \| \| \| \| \| \| \| \| \| \| --- \| --- \| --- \| --- \| --- \| --- \| --- \| --- \| --- \| --- \| \| **Variables** \| **Before PSM** \| \| \| \|  \| **After PSM** \| \| \| \| \| **COPD (n= 248)** \| **Non-COPD (n=10476)** \| **P value** \| **Standardized Difference, %** \|  \| **COPD (n=245)** \| **Non-COPD (n=965)** \| **P value** \| **Standardized Difference, %** \| \| ***Demographic characteristics*** \|  \|  \|  \|  \|  \|  \|  \|  \|  \| \| **Age, years, %** \|  \|  \|  \|  \|  \|  \|  \|  \|  \| \| Mean \| 65.4±9.2 \| 58.2±10.3 \| <0.001* \| 78.4 \|  \| 65.3±9.1 \| 64.6±9.9 \| 0.257 \| 7.2 \| \| 20-54 \| 37(14.9) \| 4063(38.8) \| <0.001* \|  \|  \| 37(15.1) \| 155(16.1) \| 0.987 \|  \| \| 55-64 \| 79(31.9) \| 3859(36.8) \|  \|  \|  \| 79(32.2) \| 307(31.8) \|  \|  \| \| 65-74 \| 88(35.5) \| 1917(18.3) \|  \|  \|  \| 87(35.5) \| 338(35.0) \|  \|  \| \| 75- \| 44(17.7) \| 617(5.9) \|  \|  \|  \| 42(17.1) \| 165(17.1) \|  \|  \| \| **Male,%** \| 189(76.2) \| 8082(77.2) \| 0.811 \| 2.2 \|  \| 186(75.9) \| 724(75.0) \| 0.773 \| 2 \| \| **CAD,%** \|  \|  \|  \|  \|  \|  \|  \|  \|  \| \| CCS \| 28(11.3) \| 1552(14.8) \| 0.122 \| 5.3 \|  \| 25(10.2) \| 96(9.9) \| 0.943 \| 0.6 \| \| ACS \| 220(88.7) \| 8923(85.2) \|  \|  \|  \| 220(89.8) \| 869(90.1) \|  \|  \| \| STEMI \| 46(18.5) \| 1847(20.6) \| 0.552 \|  \|  \| 46(18.5) \| 193(20.0) \| 0.734 \|  \| \| NSTEMI \| 37(14.9) \| 1475(14.1) \| ref \|  \|  \| 37(14.9) \| 121(12.5) \| ref \|  \| \| UAP \| 137(55.2) \| 5601(53.4) \| ref \|  \|  \| 137(55.2) \| 555(57.5) \| ref \|  \| \| ***Coexisting conditions, %*** \|  \|  \|  \|  \|  \|  \|  \|  \|  \| \| Hypertension \| 163(65.7) \| 6743(64.4) \| 0.659 \| 2.9 \|  \| 161(65.7) \| 603(62.5) \| 0.35 \| 7 \| \| Dyslipidemia \| 176(70.9) \| 7036(67.2) \| 0.207 \| 8.4 \|  \| 174(71.0) \| 675(69.9) \| 0.743 \| 1.7 \| \| Diabetes Mellitus \| 73(29.4) \| 3169(30.3) \| 0.782 \| 1.8 \|  \| 72(29.4) \| 277(28.7) \| 0.833 \| 1.9 \| \| Renal Dysfunction \| 15(6.0) \| 354(3.4) \| 0.023* \| 11.2 \|  \| 14(5.7) \| 63(6.5) \| 0.641 \| 3.6 \| \| Smoker \| 146(58.9) \| 5994(57.2) \| 0.603 \| 2 \|  \| 141(57.6) \| 549(56.9) \| 0.852 \| 0.7 \| \| Cerebrovascular Diseases \| 41(16.5) \| 1108(10.6) \| 0.003* \| 16 \|  \| 39(15.9) \| 157(16.3) \| 0.894 \| 2.3 \| \| Previous Myocardial Infarction \| 89(35.9) \| 3758(35.9) \| 0.996 \| 0 \|  \| 87(35.5) \| 339(35.1) \| 0.911 \| 0.9 \| \| Previous CABG \| 11(4.4) \| 426(4.1) \| 0.771 \| 1.8 \|  \| 11(4.5) \| 40(4.1) \| 0.81 \| 1.8 \| \| Previous PCI \| 52(20.9) \| 2596(24.5) \| 0.169 \| 9.3 \|  \| 52(21.2) \| 193(20.0) \| 0.67 \| 3.5 \| \| Peripheral Vascular Diseases \| 31(12.5) \| 781(7.5) \| 0.003* \| 19 \|  \| 16(6.5) \| 49(5.1) \| 0.368 \| 3.4 \| \| ***Lab test*** \|  \|  \|  \|  \|  \|  \|  \|  \|  \| \| Creatine, umol/l \| 78.8±19.3 \| 75.6±15.9 \| 0.011* \|  \|  \| 78.5±19.1 \| 77.7±17.9 \| 0.523 \|  \| \| Urine acid, umol/l \| 344±91.3 \| 342.2±84.9 \| 0.736 \|  \|  \| 343.8±89.8 \| 339.0±86.2 \| 0.44 \|  \| \| HbA1C, % \| 6.7±1.2 \| 6.6±1.2 \| 0.211 \|  \|  \| 6.7±1.2 \| 6.6±1.2 \| 0.216 \|  \| \| LDL-C, mmol/l \| 2.5±0.8 \| 2.5±0.9 \| 0.353 \|  \|  \| 2.5±0.8 \| 2.5±0.9 \| 0.602 \|  \| \| Albumin, g/l \| 41.3±4.3 \| 42.9±4.1 \| 0.001* \|  \|  \| 41.4±4.2 \| 42.3±4.2 \| 0.002* \|  \| \| LVEF, % \| 61.4±7.6 \| 62.8±7.4 \| 0.25 \|  \|  \| 61.5±7.3 \| 62.3±7.6 \| 0.17 \|  \| \| ***Angiographic and Procedural details*** \| \|  \|  \|  \|  \|  \|  \|  \|  \| \| Left Main involved, % \| 6(2.4) \| 340(3.2) \| 0.467 \|  \|  \| 6(2.4) \| 39(4.0) \| 0.14 \|  \| \| LAD involved, % \| 178(71.8) \| 7523(71.8) \| 0.99 \|  \|  \| 175(71.4) \| 719(74.5) \| 0.103 \|  \| \| RCA involved, % \| 142(57.3) \| 5604(53.5) \| 0.24 \|  \|  \| 139(56.7) \| 532(55.1) \| 0.06 \|  \| \| LCX involved, % \| 123(49.6) \| 5303(50.6) \| 0.75 \|  \|  \| 120(49.0) \| 484(50.2) \| 0.396 \|  \| \| TVD, % \| 90(36.3) \| 4130(39.4) \| 0.67 \|  \|  \| 86(35.1) \| 369(38.2) \| 0.67 \|  \| \| *: p ＜0.05； \|  \|  \|  \|  \|  \|  \|  \|  \|  \| \| PFT = Pulmonary Function Testing; PSM = Propensity Score Matching; COPD = Chronic Occlusion Pulmonary Disease; CAD = Coronary Artery Disease; CABG = Coronary Artery Bypass Graft ; PCI = Percutaneous Coronary Intervention ; HbA1C = Hemoglobin A1C; LDL-C = Low-density Lipoprotein Cholesterol; LVEF = Left Ventricular Eject Fraction; LAD = Left Anterior Descending; RCA = Right Coronary Artery ; LCX = Left Circumflex; TVD = Three Vessel Disease. \| \| \| \| \| \| \| \| \| \| \|  \|  \| **Supplemental Table S2. Baseline characteristics before and after Propensity-Score Matching in PFT Cohort** \| \| \| \| \| \| \| \| \| \| \| --- \| --- \| --- \| --- \| --- \| --- \| --- \| --- \| --- \| --- \| \| **Variables** \| **Before PSM** \| \| \| \|  \| **After PSM** \| \| \| \| \| **COPD**  **(n= 301)** \| **Non-COPD (n=1318)** \| **P** \| **Standardized Difference, %** \|  \| **COPD (n=301)** \| **Non-COPD (n=586)** \| **P** \| **Standardized Difference, %** \| \| ***Demographic characteristics*** \|  \|  \|  \|  \|  \|  \|  \|  \|  \| \| **Age, years, %** \|  \|  \|  \|  \|  \|  \|  \|  \|  \| \| Mean \| 62.8±9.3 \| 59.1±10.5 \| <0.001* \| 36.6 \|  \| 62.8±9.3 \| 61.9±10.1 \| 0.215 \| 2.3 \| \| 20-54 \| 52 (17.4) \| 409 (31.0) \| <0.001* \|  \|  \| 52 (17.4) \| 118 (20.1) \| 0.432 \|  \| \| 55-64 \| 110 (36.5) \| 501 (38.0) \|  \|  \|  \| 110 (36.5) \| 218 (37.2) \|  \|  \| \| 65-74 \| 110 (36.5) \| 324 (24.6) \|  \|  \|  \| 110 (36.5) \| 185 (31.6) \|  \|  \| \| 75- \| 29 (9.6) \| 84 (6.4) \|  \|  \|  \| 29 (9.6) \| 65 (11.1) \|  \|  \| \| **Male,%** \| 253 (84.1)) \| 1035 (78.5) \| 0.032* \| 15.1 \|  \| 253 (84.1)) \| 482 (82.3) \| 0.5 \| 3.6 \| \| **CAD,%** \|  \|  \|  \|  \|  \|  \|  \|  \|  \| \| CCS \| 99 (32.9) \| 427 (32.4) \| 0.869 \| 1 \|  \| 99 (32.9) \| 191(32.6) \| 0.929 \| 1.1 \| \| ACS \| 202 (67.1) \| 891 (67.6) \|  \|  \|  \| 202 (67.1) \| 395 (67.4) \|  \|  \| \| STEMI \| 37 (12.3) \| 154 (11.7) \| 0.624 \|  \|  \| 37 (12.3) \| 63 (10.8) \| 0.892 \|  \| \| NSTEMI \| 5 (1.7) \| 40 (3.0) \| ref \|  \|  \| 5 (1.7) \| 12 (2.0) \| ref \|  \| \| UAP \| 161 (53.5) \| 697 (52.9) \| ref \|  \|  \| 161 (53.5) \| 320 (54.6) \| ref \|  \| \| ***Coexisting conditions, %*** \|  \|  \|  \|  \|  \|  \|  \|  \|  \| \| Hypertension \| 160 (53.2) \| 776 (58.9) \| 0.07 \| 11.4 \|  \| 160 (53.2) \| 308 (52.6) \| 0.866 \| 2 \| \| Dyslipidemia \| 121 (40.2) \| 574 (43.6) \| 0.289 \| 6.8 \|  \| 121 (40.2) \| 230 (39.2) \| 0.784 \| 3.4 \| \| Diabetes Mellitus \| 93 (30.9) \| 357 (27.1) \| 0.183 \| 8.2 \|  \| 93 (30.9) \| 165 (28.2) \| 0.395 \| 4.7 \| \| Renal Dysfunction \| 20 (6.6) \| 91 (6.9) \| 0.872 \| 1 \|  \| 20 (6.6) \| 36 (6.1) \| 0.771 \| 2.7 \| \| Smoker \| 199 (66.1) \| 753 (57.1) \| 0.004* \| 18.9 \|  \| 199 (66.1) \| 396 (67.6) \| 0.66 \| 4.6 \| \| Cerebrovascular Diseases \| 25 (8.3) \| 99 (7.5) \| 0.64 \| 2.9 \|  \| 25 (8.3) \| 48 (8.2) \| 0.953 \| 0.6 \| \| Previous Myocardial Infarction \| 18 (6.0) \| 65 (4.9) \| 0.457 \| 4.4 \|  \| 18 (6.0) \| 35 (6.0) \| 0.997 \| 1.4 \| \| Previous CABG \| 0 \| 6 (0.5) \| 0.241 \| - \|  \| 0 \| 0 \| - \| - \| \| Previous PCI \| 1 (0.3) \| 4 (0.3) \| 0.935 \| 0.5 \|  \| 1 (0.3) \| 3 (0.5) \| 0.705 \| 2.9 \| \| Peripheral Vascular Diseases \| 11 (3.7) \| 35 (2.7) \| 0.347 \| 19.2 \|  \| 11 (3.7) \| 23 (3.9) \| 0.843 \| 0.9 \| \| ***Lab test*** \|  \|  \|  \|  \|  \|  \|  \|  \|  \| \| Creatine, umol/l \| 80.4±18.2 \| 85.9±177.2 \| 0.589 \|  \|  \| 80.4±18.2 \| 93.0±264.5 \| 0.409 \|  \| \| Urine acid, umol/l \| 348.2±88.6 \| 355.3±85.0 \| 0.195 \|  \|  \| 348.2±88.6 \| 354.3±81.4 \| 0.305 \|  \| \| HbA1C, % \| 6.6±1.3 \| 6.6±1.4 \| 0.82 \|  \|  \| 6.6±1.3 \| 6.6±1.3 \| 0.862 \|  \| \| LDL-C, mmol/l \| 2.5±0.9 \| 2.6±0.9 \| 0.279 \|  \|  \| 2.5±0.9 \| 2.6±0.9 \| 0.632 \|  \| \| Albumin, g/l \| 40.9±3.7 \| 41.6±4.0 \| 0.005* \|  \|  \| 40.9±3.7 \| 41.2±4.0 \| 0.321 \|  \| \| ***Angiographic and Procedural details*** \| \|  \|  \|  \|  \|  \|  \|  \|  \| \| Left Main involved, % \| 15 (5.0) \| 47 (3.6) \| 0.248 \|  \|  \| 15 (5.0) \| 23 (3.9) \| 0.461 \|  \| \| LAD involved, % \| 187 (62.1) \| 738 (56.0) \| 0.052 \|  \|  \| 187 (62.1) \| 330 (56.3) \| 0.096 \|  \| \| RCA involved, % \| 113 (37.5) \| 499 (37.9) \| 0.918 \|  \|  \| 113 (37.5) \| 227 (38.7) \| 0.729 \|  \| \| LCX involved, % \| 86 (28.6) \| 346 (26.3) \| 0.412 \|  \|  \| 86 (28.6) \| 175 (29.9) \| 0.689 \|  \| \| TVD, % \| 12 (4.0) \| 53 (4.0) \| 0.978 \|  \|  \| 12 (4.0) \| 25 (4.3) \| 0.844 \|  \| \| ***Cardiac Function and Arrythmia*** \|  \|  \|  \|  \|  \|  \|  \|  \|  \| \| LVEF, % \| 67.0±9.1 \| 66.4±8.8 \| 0.462 \|  \|  \| 67.0±9.1 \| 66.6±9.0 \| 0.655 \|  \| \| NYHA I Class \| 259 (86.0) \| 1126 (85.4) \| 0.604 \|  \|  \| 259 (86.0) \| 493 (84.1) \| 0.721 \|  \| \| NYHA II Class \| 33 (11.0) \| 160 (12.1) \| ref \|  \|  \| 33 (11.0) \| 76 (13.0) \| ref \|  \| \| NYHA III Class \| 8 (2.7) \| 23 (1.7) \| ref \|  \|  \| 8 (2.7) \| 13 (2.2) \| ref \|  \| \| NYHA IV Class \| 1 (0.3) \| 9 (0.7) \| ref \|  \|  \| 1 (0.3) \| 4 (0.7) \| ref \|  \| \| Atrial Fibrillation \| 3 (1.0) \| 17 (1.3) \| 0.678 \|  \|  \| 3 (1.0) \| 9 (1.5) \| 0.51 \|  \| \| Sinus Bradycardia \| 1 (0.3) \| 1 (0.07) \| 0.253 \|  \|  \| 1 (0.3) \| 1 (0.17) \| 0.631 \|  \| \| Atrioventricular Block \| 1 (0.3) \| 1 (0.07) \| 0.253 \|  \|  \| 1 (0.3) \| 0 \| - \|  \| \| Ventricular Arrythmia \| 0 \| 2 (0.15) \| - \|  \|  \| 0 \| 2 (0.34) \| - \|  \| \| ***Pulmonary Function Test*** \|  \|  \|  \|  \|  \|  \|  \|  \|  \| \| FEV1/FVC, % \| 61.6±7.9 \| 79.6±5.3 \| <0.001* \|  \|  \| 61.6±7.9 \| 79.2±5.5 \| <0.001* \|  \| \| FEV1, %pred \| 74.4±17.5 \| 91.2±13.2 \| <0.001* \|  \|  \| 74.4±17.5 \| 91.7±14.4 \| <0.001* \|  \| \| ***Anticogulation therapy, %*** \| 3 (1.0) \| 17 (1.3) \| 0.678 \|  \|  \| 3 (1.0) \| 9 (1.5) \| 0.51 \|  \| \| *: p ＜0.05； \|  \|  \|  \|  \|  \|  \|  \|  \|  \| \| PFT = Pulmonary Function Testing; PSM = Propensity Score Matching; COPD = Chronic Occlusion Pulmonary Disease; CAD = Coronary Artery Disease; CABG = Coronary Artery Bypass Graft ; PCI = Percutaneous Coronary Intervention ; HbA1C = Hemoglobin A1C; LDL-C = Low-density Lipoprotein Cholesterol; LVEF = Left Ventricular Eject Fraction; LAD = Left Anterior Descending; RCA = Right Coronary Artery ; LCX = Left Circumflex; TVD = Three Vessel Disease; FEV1 = Forced Expiratory Volume in the first second; FVC = Forced Vital Capacity. \| \| \| \| \| \| \| \| \| \| \|  \| |  |  |  |  |  |  |  |  |  |
| --- | --- | --- | --- | --- | --- | --- | --- | --- | --- | --- | --- | --- | --- | --- | --- | --- | --- | --- | --- | --- | --- | --- | --- | --- | --- | --- | --- | --- | --- | --- | --- | --- | --- | --- | --- | --- | --- | --- | --- | --- | --- | --- | --- | --- | --- | --- | --- | --- | --- | --- | --- | --- | --- | --- | --- | --- | --- | --- | --- | --- | --- | --- | --- | --- | --- | --- | --- | --- | --- | --- | --- | --- | --- | --- | --- | --- | --- | --- | --- | --- | --- | --- | --- | --- | --- | --- | --- | --- | --- | --- | --- | --- | --- | --- | --- | --- | --- | --- | --- | --- | --- | --- | --- | --- | --- | --- | --- | --- | --- | --- | --- | --- | --- | --- | --- | --- | --- | --- | --- | --- | --- | --- | --- | --- | --- | --- | --- | --- | --- | --- | --- | --- | --- | --- | --- | --- | --- | --- | --- | --- | --- | --- | --- | --- | --- | --- | --- | --- | --- | --- | --- | --- | --- | --- | --- | --- | --- | --- | --- | --- | --- | --- | --- | --- | --- | --- | --- | --- | --- | --- | --- | --- | --- | --- | --- | --- | --- | --- | --- | --- | --- | --- | --- | --- | --- | --- | --- | --- | --- | --- | --- | --- | --- | --- | --- | --- | --- | --- | --- | --- | --- | --- | --- | --- | --- | --- | --- | --- | --- | --- | --- | --- | --- | --- | --- | --- | --- | --- | --- | --- | --- | --- | --- | --- | --- | --- | --- | --- | --- | --- | --- | --- | --- | --- | --- | --- | --- | --- | --- | --- | --- | --- | --- | --- | --- | --- | --- | --- | --- | --- | --- | --- | --- | --- | --- | --- | --- | --- | --- | --- | --- | --- | --- | --- | --- | --- | --- | --- | --- | --- | --- | --- | --- | --- | --- | --- | --- | --- | --- | --- | --- | --- | --- | --- | --- | --- | --- | --- | --- | --- | --- | --- | --- | --- | --- | --- | --- | --- | --- | --- | --- | --- | --- | --- | --- | --- | --- | --- | --- | --- | --- | --- | --- | --- | --- | --- | --- | --- | --- | --- | --- | --- | --- | --- | --- | --- | --- | --- | --- | --- | --- | --- | --- | --- | --- | --- | --- | --- | --- | --- | --- | --- | --- | --- | --- | --- | --- | --- | --- | --- | --- | --- | --- | --- | --- | --- | --- | --- | --- | --- | --- | --- | --- | --- | --- | --- | --- | --- | --- | --- | --- | --- | --- | --- | --- | --- | --- | --- | --- | --- | --- | --- | --- | --- | --- | --- | --- | --- | --- | --- | --- | --- | --- | --- | --- | --- | --- | --- | --- | --- | --- | --- | --- | --- | --- | --- | --- | --- | --- | --- | --- | --- | --- | --- | --- | --- | --- | --- | --- | --- | --- | --- | --- | --- | --- | --- | --- | --- | --- | --- | --- | --- | --- | --- | --- | --- | --- | --- | --- | --- | --- | --- | --- | --- | --- | --- | --- | --- | --- | --- | --- | --- | --- | --- | --- | --- | --- | --- | --- | --- | --- | --- | --- | --- | --- | --- | --- | --- | --- | --- | --- | --- | --- | --- | --- | --- | --- | --- | --- | --- | --- | --- | --- | --- | --- | --- | --- | --- | --- | --- | --- | --- | --- | --- | --- | --- | --- | --- | --- | --- | --- | --- | --- | --- | --- | --- | --- | --- | --- | --- | --- | --- | --- | --- | --- | --- | --- | --- | --- | --- | --- | --- | --- | --- | --- | --- | --- | --- | --- | --- | --- | --- | --- | --- | --- | --- | --- | --- | --- | --- | --- | --- | --- | --- | --- | --- | --- | --- | --- | --- | --- | --- | --- | --- | --- | --- | --- | --- | --- | --- | --- | --- | --- | --- | --- | --- | --- | --- | --- | --- | --- | --- | --- | --- | --- | --- | --- | --- | --- | --- | --- | --- | --- | --- | --- | --- | --- | --- | --- | --- | --- | --- | --- | --- | --- | --- | --- | --- | --- | --- | --- | --- | --- | --- | --- | --- | --- | --- | --- | --- | --- | --- | --- | --- | --- | --- | --- | --- | --- | --- | --- | --- | --- | --- | --- | --- | --- | --- | --- | --- | --- | --- | --- | --- | --- | --- | --- | --- | --- | --- | --- | --- | --- | --- | --- | --- | --- | --- | --- | --- | --- | --- | --- | --- | --- | --- | --- | --- | --- | --- | --- | --- | --- | --- | --- | --- | --- | --- | --- | --- | --- | --- | --- | --- | --- | --- | --- | --- | --- | --- | --- | --- | --- | --- | --- | --- | --- | --- | --- | --- | --- | --- | --- | --- | --- | --- | --- | --- | --- | --- | --- | --- | --- | --- | --- | --- | --- | --- | --- | --- | --- | --- | --- | --- | --- | --- | --- | --- | --- | --- | --- | --- | --- | --- | --- | --- | --- | --- | --- | --- | --- | --- | --- | --- | --- | --- | --- | --- | --- | --- | --- | --- | --- | --- | --- | --- | --- | --- | --- | --- | --- | --- | --- | --- | --- | --- | --- | --- | --- | --- | --- | --- | --- | --- | --- | --- | --- | --- | --- | --- | --- | --- | --- | --- | --- | --- | --- | --- | --- | --- | --- | --- | --- | --- | --- | --- | --- | --- | --- | --- | --- | --- | --- | --- | --- | --- | --- | --- | --- | --- | --- | --- | --- | --- | --- | --- | --- | --- | --- | --- | --- | --- | --- | --- | --- | --- | --- | --- | --- | --- | --- | --- | --- | --- | --- | --- | --- | --- | --- | --- | --- | --- | --- | --- | --- | --- | --- | --- | --- | --- | --- | --- | --- | --- | --- | --- | --- | --- | --- | --- | --- | --- | --- | --- | --- | --- | --- | --- | --- | --- | --- | --- | --- | --- | --- | --- | --- | --- | --- | --- | --- | --- | --- | --- | --- | --- | --- | --- | --- | --- | --- | --- | --- | --- | --- | --- | --- | --- | --- | --- | --- | --- | --- | --- | --- | --- | --- | --- | --- | --- | --- | --- | --- | --- | --- | --- | --- | --- | --- | --- | --- | --- | --- | --- | --- | --- | --- | --- | --- | --- | --- | --- | --- | --- | --- | --- | --- | --- | --- | --- | --- | --- | --- | --- | --- | --- | --- | --- | --- | --- | --- | --- | --- | --- | --- | --- | --- | --- | --- | --- | --- | --- | --- | --- | --- | --- | --- | --- | --- | --- | --- | --- | --- | --- | --- | --- | --- | --- | --- | --- | --- | --- | --- | --- | --- | --- | --- | --- | --- | --- | --- | --- | --- | --- | --- | --- | --- | --- | --- | --- | --- | --- | --- | --- | --- | --- | --- | --- | --- |

|  |  |  |  |  |  |  |  |  |  |  |  |  |  |
| --- | --- | --- | --- | --- | --- | --- | --- | --- | --- | --- | --- | --- | --- |
| **Supplemental Table S3. Effect modification of age on long-term outcomes of patients after PCI with or without COPD in PFT cohort** | | | | | | | | | | | | | |
|  | **COPD** | |  | **non-COPD** | |  | **Crude** | | **P value for interaction** |  | **Adjusted** | | **Adjusted P value for interaction** |
| **Outcomes** | Total | Events, % |  | Total | Events, % |  | Hazard Ratio ( 95% Confidence Interval ) | **P** |  |  | Adjusted Hazard Ratio ( 95%Confidence Interval ) | **P** |  |
| **MACE** |  |  |  |  |  |  |  |  |  |  |  |  |  |
| Age Q3(>= 75 yrs) | 29 | 7 (24.1) |  | 65 | 5 (7.7) |  | 3.818 (1.10-13.29) | 0.027† | 0.068* |  | 3.772 (1.07-13.30) | 0.039† | 0.071* |
| Age Q0-Q2 | 272 | 37 (13.6) |  | 521 | 58 (11.1) |  | 1.257 (0.81-1.95) | 0.309 |  |  | 1.270 (0.82-1.98) | 0.29 |  |
| Age Q0(20-54, yrs) | 52 | 11 (21.2) |  | 118 | 7 (5.9) |  | 4.254 (1.55-11.72) | 0.003† | 0.02† |  | 4.295 (1.56-11.86) | 0.005† | 0.019† |
| Age Q1-Q3 | 249 | 33 (13.3) |  | 468 | 56 (12.0) |  | 1.124 (0.71-1.78) | 0.619 |  |  | 1.129 (0.71-1.79) | 0.607 |  |
| Age Q1(55-64, yrs) | 110 | 13 (11.8) |  | 218 | 26 (11.9) |  | 0.99 (0.49-2.01) | 0.977 |  |  | 1.013 (0.50-2.07) | 0.971 |  |
| Age Q2(65-74, yrs) | 110 | 13 (11.8) |  | 185 | 25 (13.5) |  | 0.858 (0.42-1.76) | 0.674 |  |  | 0.851 (0.42-1.75) | 0.661 |  |
| **Death** |  |  |  |  |  |  |  |  |  |  |  |  |  |
| Age Q3(>= 75 yrs) | 29 | 3 (10.3) |  | 65 | 6 (9.2) |  | 1.135 (0.26-4.89) | 0.865 | 0.783 |  | 1.030 (0.23-4.64) | 0.969 | 0.719 |
| Age Q0-Q2 | 272 | 9 (3.3) |  | 521 | 6 (1.2) |  | 2.937 (1.03-8.34) | 0.034† |  |  | 3.059 (1.07-8.73) | 0.036† |  |
| Age Q0(20-54, yrs) | 52 | 0 |  | 118 | 0 |  | NA | NA | 0.449 |  | NA | NA | 0.466 |
| Age Q1-Q3 | 249 | 12 (4.8) |  | 468 | 12 (2.6) |  | 1.924 (0.85-4.35) | 0.11 |  |  | 1.920 (0.85-4.35) | 0.118 |  |
| Age Q1(55-64, yrs) | 110 | 2 (1.8) |  | 218 | 2 (0.9) |  | 2.000 (0.28-14.39) | 0.483 |  |  | 2.026 (0.28-14.86) | 0.487 |  |
| Age Q2(65-74, yrs) | 110 | 7 (6.4) |  | 185 | 4 (2.2) |  | 3.075 (0.88-10.76) | 0.079* |  |  | 2.994 (0.84-10.64) | 0.09* |  |
| **Cardiac Death** |  |  |  |  |  |  |  |  |  |  |  |  |  |
| Age Q3(>= 75 yrs) | 29 | 1(3.4) |  | 65 | 1 (1.5) |  | 2.286 (0.14-37.86) | 0.564 | 0.158 |  | 2.364 (0.14-41.28) | 0.556 | 0.18 |
| Age Q0-Q2 | 272 | 0 |  | 521 | 3 (0.6) |  | NA | 0.21 |  |  | NA | 0.994 |  |
| Age Q0(20-54, yrs) | 52 | 0 |  | 118 | 0 |  | NA | NA | 0.743 |  | NA | NA | 0.715 |
| Age Q1-Q3 | 249 | 1 (0.4) |  | 468 | 4 (0.9) |  | 0.468 (0.05-4.21) | 0.488 |  |  | 0.467 (0.05-4.22) | 0.497 |  |
| Age Q1(55-64, yrs) | 110 | 0 |  | 218 | 1 (0.5) |  | NA | 0.477 |  |  | NA | 1 |  |
| Age Q2(65-74, yrs) | 110 | 0 |  | 185 | 2 (1.1) |  | NA | 0.274 |  |  | NA | 0.996 |  |
| **Myocardial Infarction** |  |  |  |  |  |  |  |  |  |  |  |  |  |
| Age Q3(>= 75 yrs) | 29 | 0 |  | 65 | 0 |  | NA | NA | 0.872 |  | NA | NA | 0.853 |
| Age Q0-Q2 | 272 | 3 (1.1) |  | 521 | 4 (0.8) |  | 1.441 (0.32-6.49) | 0.632 |  |  | 1.496 (0.33-6.76) | 0.601 |  |
| Age Q0(20-54, yrs) | 52 | 1 (1.9) |  | 118 | 1 (0.8) |  | 2.294 (0.14-37.40) | 0.549 | 0.576 |  | 2.486(0.15-40.85) | 0.524 | 0.556 |
| Age Q1-Q3 | 249 | 2 (0.8) |  | 468 | 3 (0.6) |  | 1.255 (0.21-7.56) | 0.804 |  |  | 1.265 (0.21-7.67) | 0.798 |  |
| Age Q1(55-64, yrs) | 110 | 2 (1.8) |  | 218 | 1 (0.5) |  | 4.019 (0.36-44.81) | 0.222 |  |  | 4.229 (0.38-47.42) | 0.242 |  |
| Age Q2(65-74, yrs) | 110 | 0 |  | 185 | 2 (1.1) |  | NA | 0.274 |  |  | NA | 0.995 |  |
| **Revascularization** |  |  |  |  |  |  |  |  |  |  |  |  |  |
| Age Q3(>= 75 yrs) | 29 | 6 (20.7) |  | 65 | 4 (6.2) |  | 3.978 (1.028-15.392) | 0.045† | 0.117 |  | 3.914 (1.01-15.23) | 0.049† | 0.118 |
| Age Q0-Q2 | 272 | 36 (13.2) |  | 521 | 54 (10.4) |  | 1.319 (0.84-2.07) | 0.226 |  |  | 1.330 (0.85-2.10) | 0.214 |  |
| Age Q0(20-54, yrs) | 52 | 11 (21.2) |  | 118 | 7 (5.9) |  | 4.254 (1.55-11.72) | 0.003† | 0.019† |  | 4.295 (1.56-11.86) | 0.005† | 0.018† |
| Age Q1-Q3 | 249 | 31 (12.4) |  | 468 | 51 (10.9) |  | 1.163 (0.72-1.87) | 0.534 |  |  | 1.168 (0.73-1.88) | 0.523 |  |
| Age Q1(55-64, yrs) | 110 | 12 (10.9) |  | 218 | 25 (11.5) |  | 0.945 (0.46-1.96) | 0.88 |  |  | 0.917 (0.46-2.00) | 0.976 |  |
| Age Q2(65-74, yrs) | 110 | 13 (11.8) |  | 185 | 22 (11.9) |  | 0.993 (0.48-2.06) | 0.985 |  |  | 0.990 (0.48-2.06) | 0.978 |  |
| **Stroke** |  |  |  |  |  |  |  |  |  |  |  |  |  |
| Age Q3(>= 75 yrs) | 29 | 0 |  | 65 | 0 |  | NA | NA | 0.868 |  | NA | NA | 0.903 |
| Age Q0-Q2 | 272 | 3 (1.1) |  | 521 | 8 (1.5) |  | 0.715 (0.19-2.72) | 0.621 |  |  | 0.734 (0.19-2.80) | 0.651 |  |
| Age Q0(20-54, yrs) | 52 | 0 |  | 118 | 0 |  | NA | NA | 0.804 |  | NA | NA | 0.778 |
| Age Q1-Q3 | 249 | 3 (1.2) |  | 468 | 8 (1.7) |  | 0.701 (0.18-2.67) | 0.601 |  |  | 0.702 (0.18-2.70) | 0.605 |  |
| Age Q1(55-64, yrs) | 110 | 1 (0.9) |  | 218 | 6 (2.8) |  | 0.324 (0.04-2.73) | 0.275 |  |  | 0.337 (0.04-2.84) | 0.317 |  |
| Age Q2(65-74, yrs) | 110 | 2 (1.8) |  | 185 | 2 (1.1) |  | 1.694 (0.24-12.20) | 0.601 |  |  | 1.645 (0.23-11.98) | 0.623 |  |
| Confounding factors enrolled in adjusted model: Smoker, Renal dysfunction | | | | | |  |  |  |  |  |  |  |  |
| †: p ＜0.05，*: p＜0.1; NA = Not Applicable; PFT = Pulmonary Function Testing; COPD = Chronic Occlusion Pulmonary Disease; PCI = Percutaneous Coronary Intervention. | | | | | | | | | | | |  |  |

| **Supplemental Table S4. Long-term outcomes after PCI in PFT Cohort according to various GOLD levels** | | | | | | | | | | | | | | | | |
| --- | --- | --- | --- | --- | --- | --- | --- | --- | --- | --- | --- | --- | --- | --- | --- | --- |
|  | **Total (n=301)** |  | **GOLD I (n=78)** |  |  | **GOLD II (n=129)** |  |  |  | **GOLD III (n=34)** |  |  |  | **GOLD IV (n=60)** |  |  |
| **Endpoints** | **No. of Events** |  | **No. of Events, %** |  | **No. of Events, %** | **Harzard Ratio (95% confidence interval)** | **P** |  | **No. of Events, %** | **Harzard Ratio (95% confidence interval)** | **P** |  | **No. of Events, %** | **Harzard Ratio (95% confidence interval)** | **P** |  |
| ***Primary Endpoint*** | 44 |  | 11(14.1) |  | 17(13.2) | 0.925(0.41 to 2.09) | 0.85 |  | 7(20.6) | 1.579(0.55 to 4.50) | 0.39 |  | 9(15.0) | 1.075(0.41 to 2.79) | 0.88 |  |
| ***Components of primary endpoint*** |  |  |  |  |  |  |  |  |  |  |  |  |  |  |  |  |
| Cardiac death | 1 |  | 0 |  | 0 | **-** | **-** |  | 0 | **-** | **-** |  | 1(0.3) | **-** | **-** |  |
| Myocardial infarction | 3 |  | 0 |  | 3(2.3) | **-** | **-** |  | 0 | **-** | **-** |  | 0 | **-** | **-** |  |
| Revascularization | 42 |  | 11(14.1) |  | 16(12.4) | 0.862(0.38 to 1.97) | 0.73 |  | 7(20.6) | 1.579(0.55 to 4.50) | 0.39 |  | 8(13.3) | 0.937(0.35 to 2.50) | 0.9 |  |
| ***Device-oriented composite endpoint*** | *25* |  | 6(7.7) |  | 9(7.0) | 0.900(0.31 to 2.63) | 0.85 |  | 4(11.8) | 1.600(0.42 to 6.08) | 0.49 |  | 6(10.0) | 1.333(0.41 to 4.36) | 0.63 |  |
| ***All-cause death*** | *12* |  | 3(3.8) |  | 3(2.3) | 0.595(0.12 to 3.03) | 0.53 |  | 3(8.8) | 2.419(0.46 to 12.65) | 0.3 |  | 3(5.0) | 1.316(0.26 to 6.76) | 0.74 |  |
| ***Stroke*** | *3* |  | 0 |  | 2(1.6) | **-** | **-** |  | 0 | **-** | **-** |  | 1(1.7) | **-** | **-** |  |
| MACE: Major Adverse Cardiovascular Events; COPD: Chronic Occlusion Pulmonary Disease; PFT: Pulmonary Function Testing; PCI: Percutaneous Coronary Intervention; PSM: Propensity Score Matching; Device-oriented composiet endpoint contains Cardiac death, target vessel myocardial infarction, and target lesion revascularization; GOLD: Global Initiative for Chronic Obstructive Lung Disease | | | | | | | | | | | | | | | | |


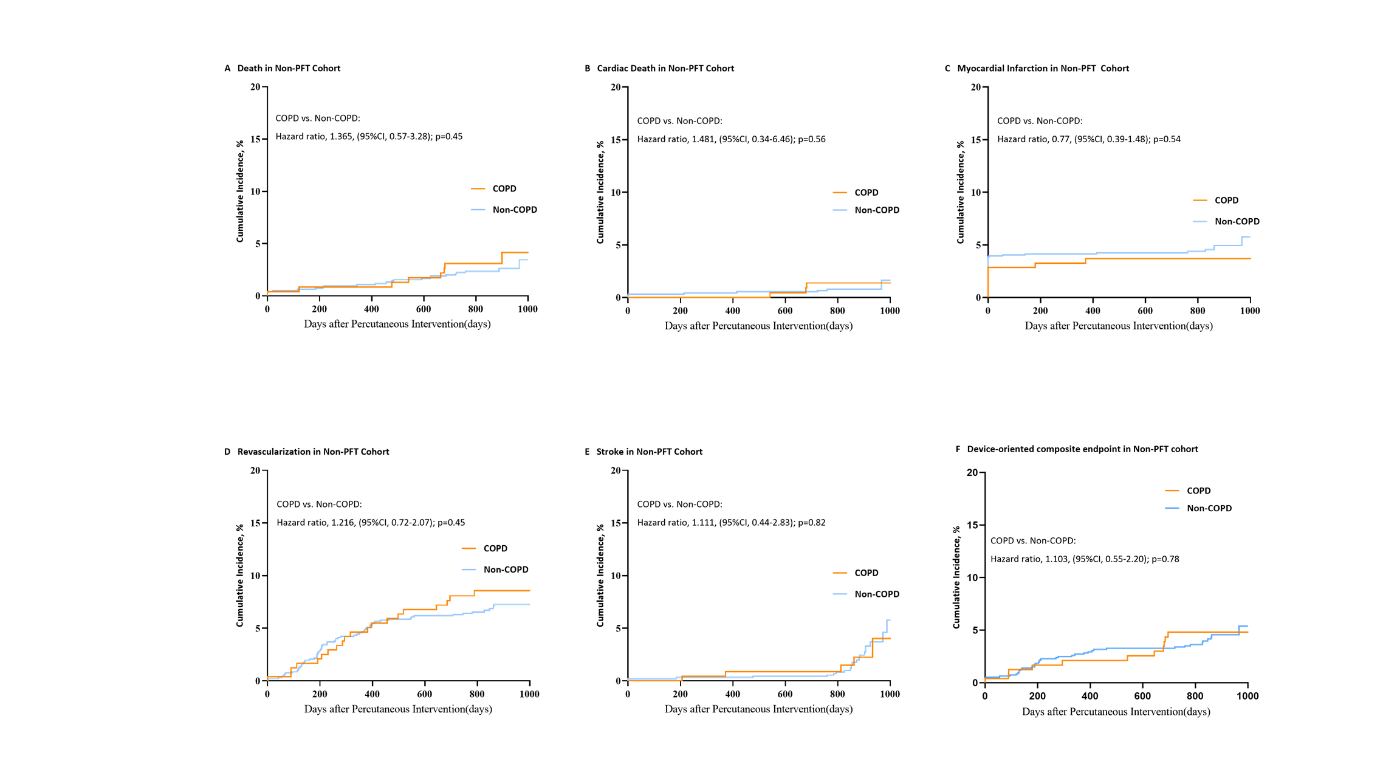


**Supplementary Figure S1.** Kaplan-Meier curves for secondary endpoint in non-PFT cohorts

**Legend:** A. Deaths; B. Cardiac Death; C. Non-fatal myocardial infarction; D. Unplanned revascularization; E. Stroke; F. DOCE. All p > 0.05


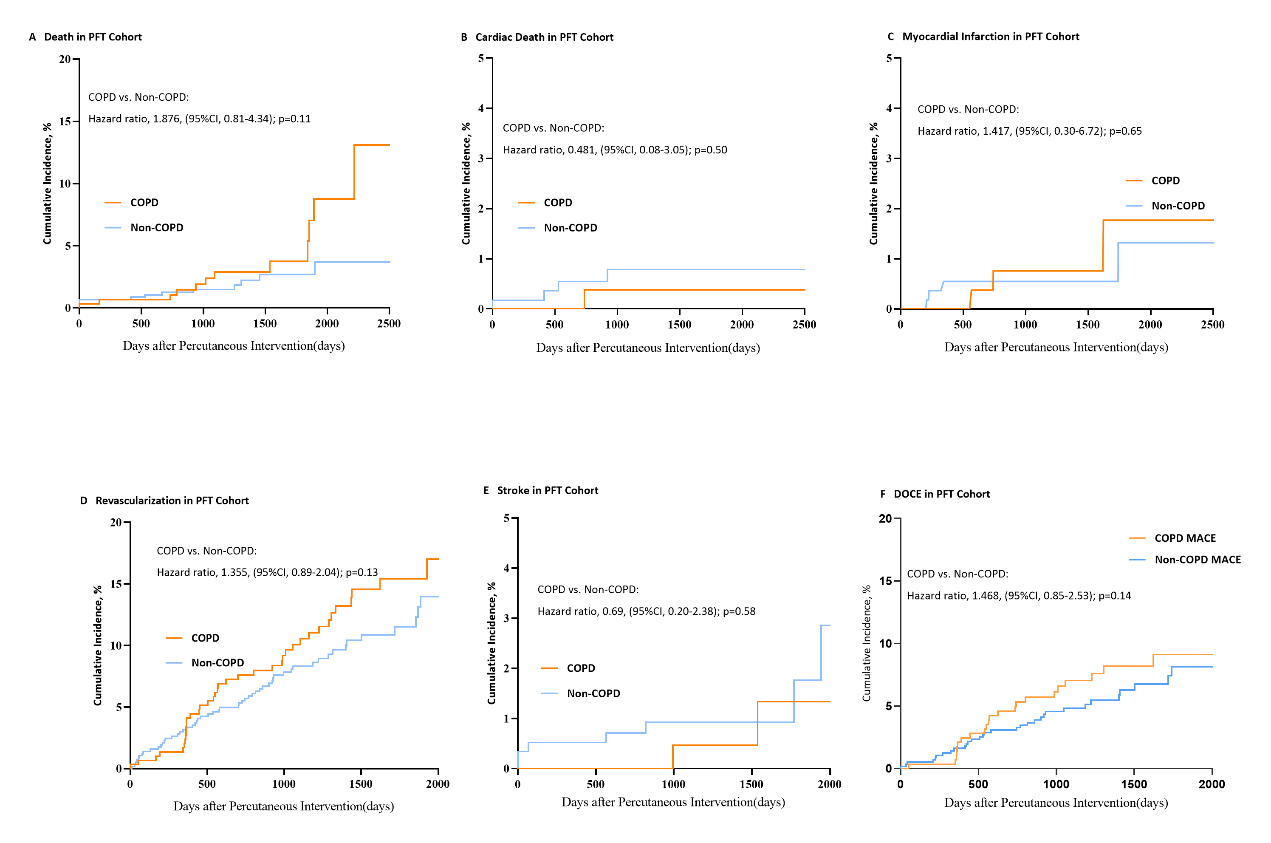


**Supplementary Figure S2.** Kaplan-Meier curves for secondary endpoint in PFT cohort

**Legend:** A. Deaths; B. Cardiac death; C. Non-fatal myocardial infarction; D. Unplanned revascularization; E. Stroke; F. DOCE. All p > 0.05.


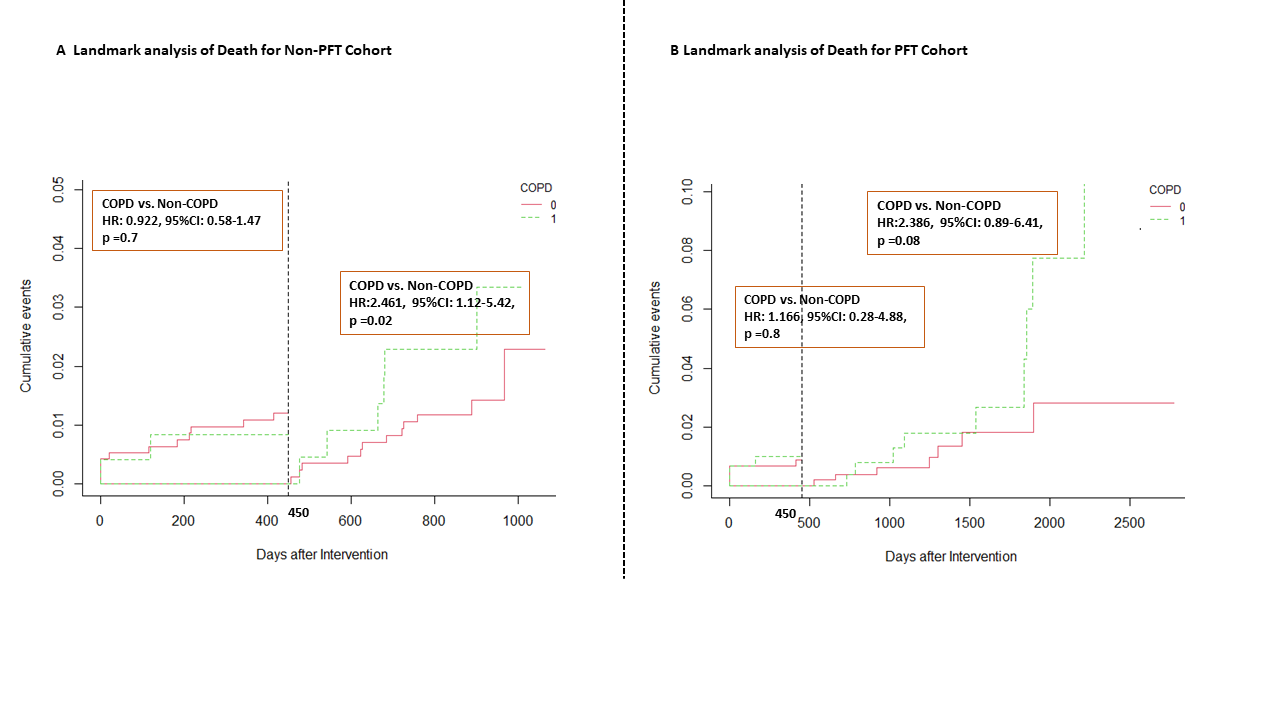


**Supplementary Figure S3.** Landmark analysis of death in double cohorts

**Legend: A.** Landmark analysis of death in non-PFT cohort; **B.** Landmark analysis of death in PFT cohort. HR = Hazard Ratio; CI = Confidence Interval


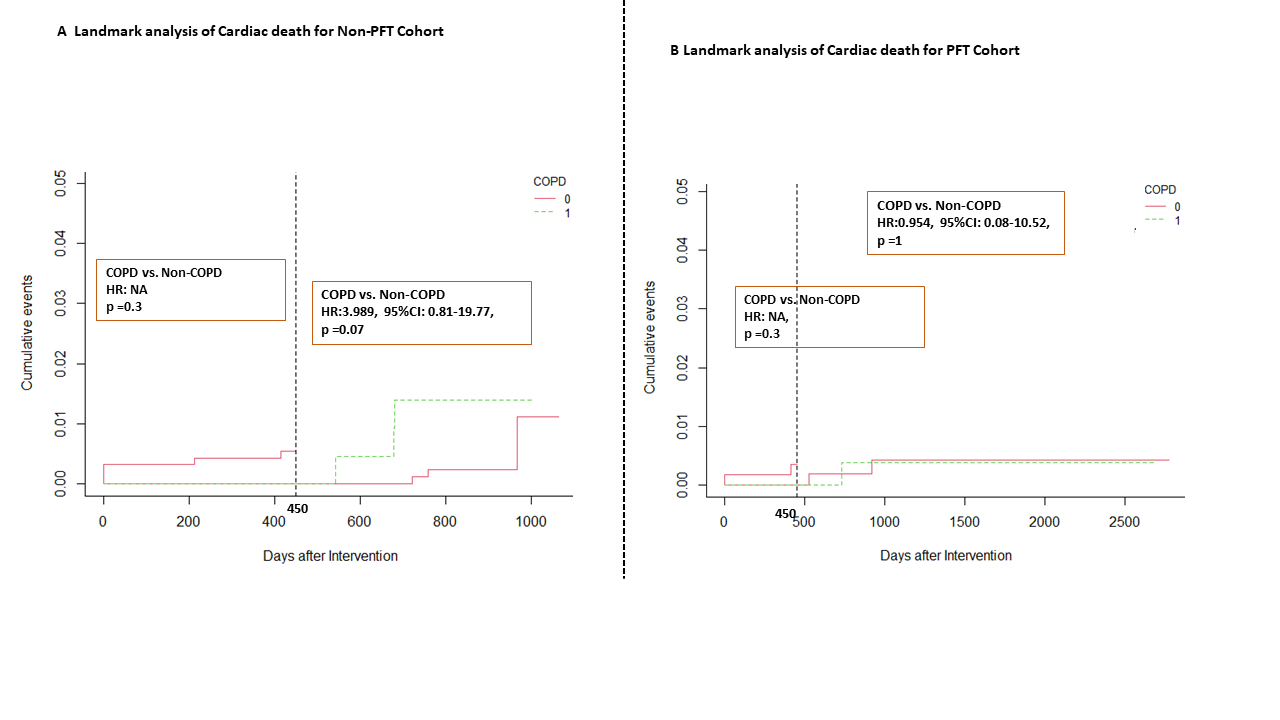
**Supplementary Figure S4.** Landmark analysis of cardiac death in double cohorts **Legend: A.** Landmark analysis of cardiac death in non-PFT cohort; **B.** Landmark analysis of cardiac death in PFT cohort. HR = Hazard Ratio; CI = Confidence Interval


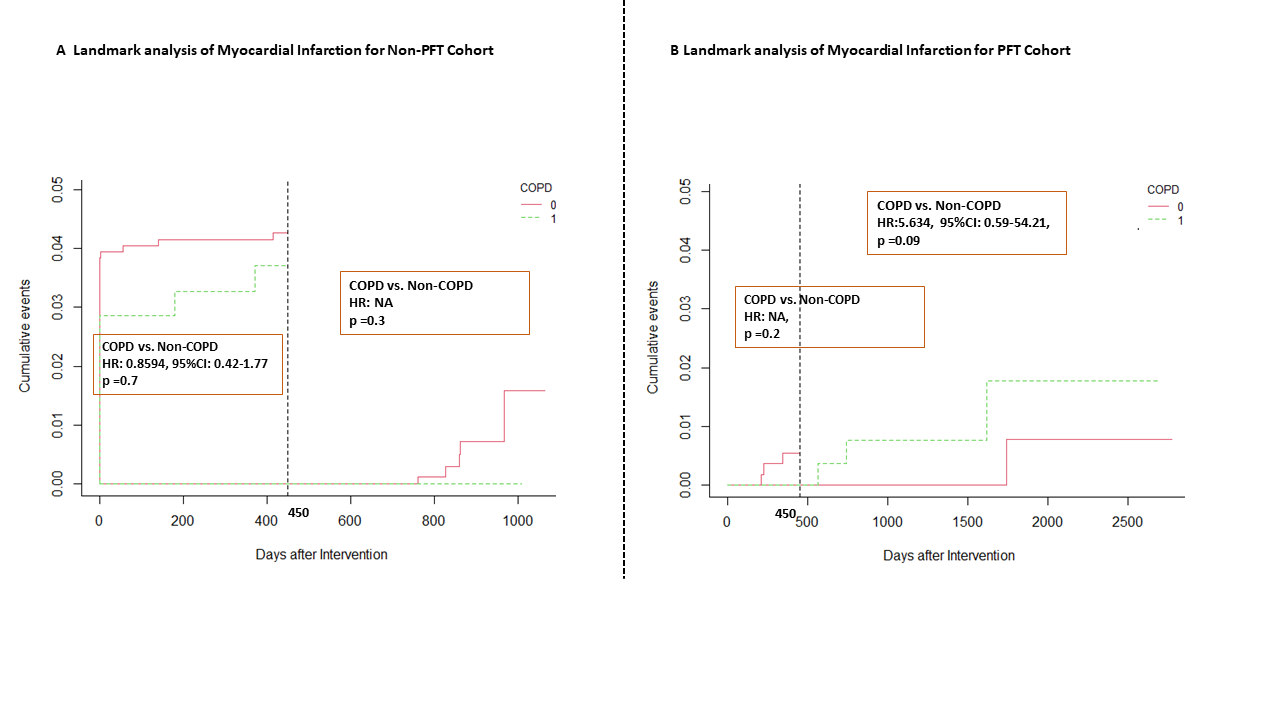


**Supplementary Figure S5.** Landmark analysis of non-fatal myocardial infarction in double cohorts

**Legend: A.** Landmark analysis of non-fatal myocardial infarction in non-PFT cohort; **B.** Landmark analysis of non-fatal myocardial infarction in PFT cohort. HR = Hazard Ratio; CI = Confidence Interval


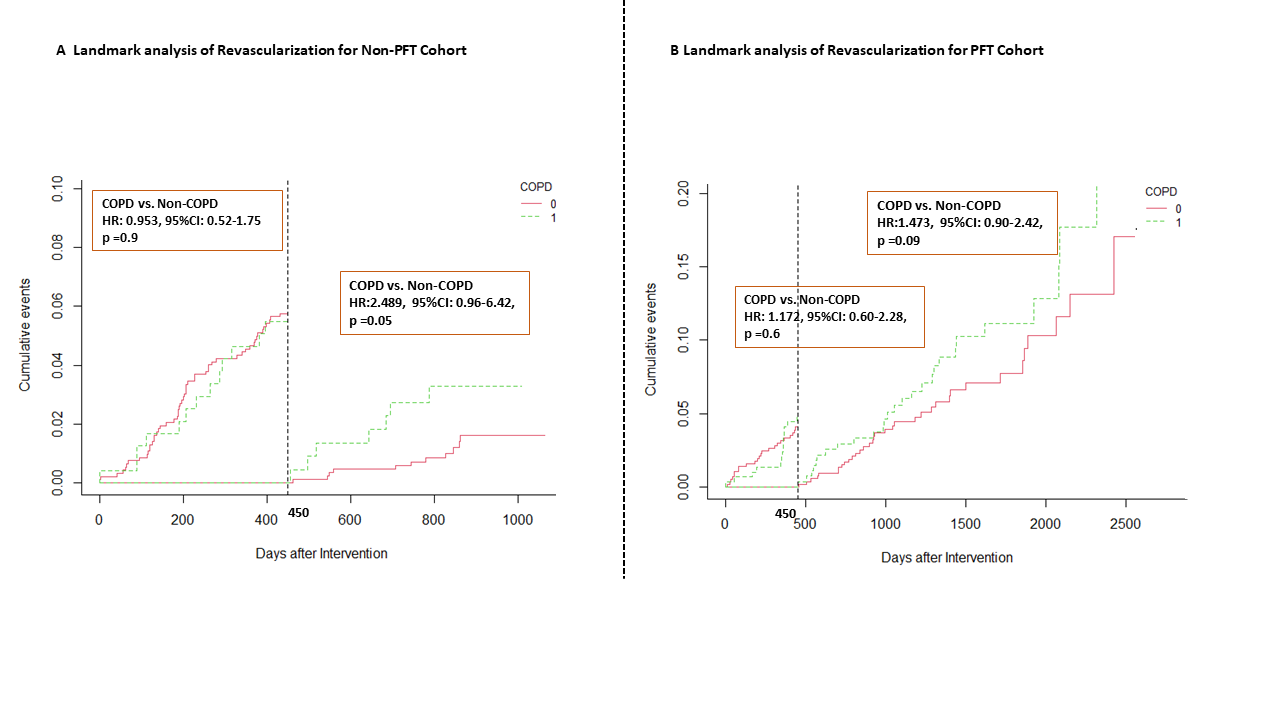


**Supplementary Figure S6.** Landmark analysis of unplanned revascularization in double cohorts

**Legend: A.** Landmark analysis of unplanned revascularization in non-PFT cohort; **B.** Landmark analysis of unplanned revascularization in PFT cohort. HR = Hazard Ratio; CI = Confidence Interval


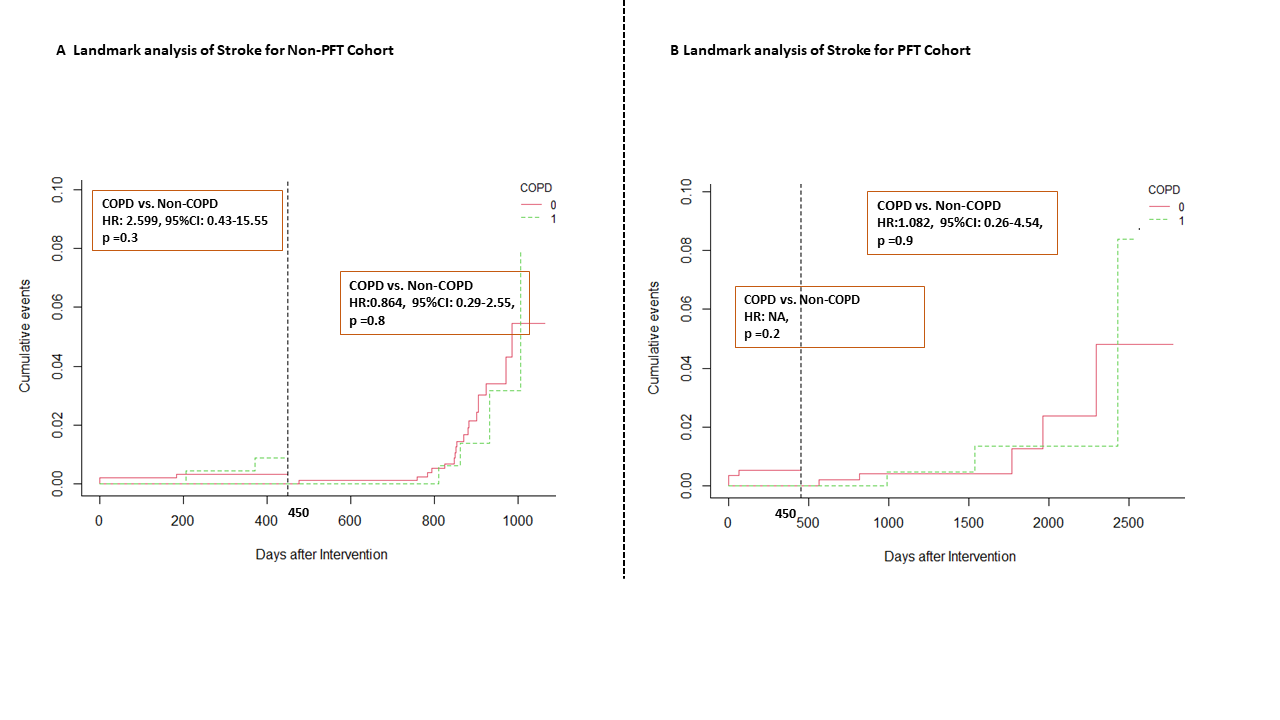


**Supplementary Figure S7.** Landmark analysis of stroke in double cohorts

**Legend: A.** Landmark analysis of stroke in non-PFT cohort; **B.** Landmark analysis of stroke in PFT cohort. HR = Hazard Ratio; CI = Confidence Interval


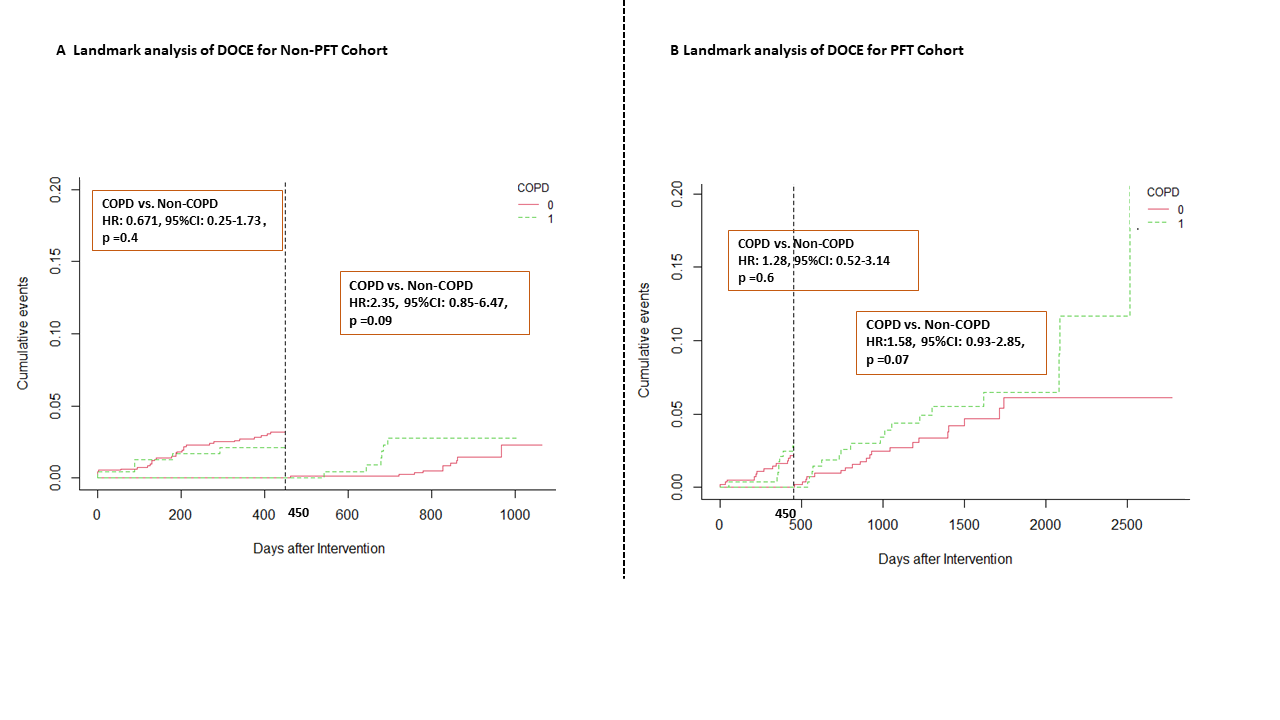


**Supplementary Figure S8.** Landmark analysis of DOCE in double cohorts

**Legend: A.** Landmark analysis of stroke in non-PFT cohort; **B.** Landmark analysis of stroke in PFT cohort. HR = Hazard Ratio; CI = Confidence Interval
